# IDENTIFYING PROFILES OF STROKE PATIENTS BENEFITTING FROM ADDITIONAL TRAINING: A LATENT CLASS ANALYSIS APPROACH

**DOI:** 10.2340/jrm.v56.22141

**Published:** 2024-02-21

**Authors:** Kohei IKEDA,, Takao KANEKO, Junya UCHIDA, Takuto NAKAMURA, Taisei TAKEDA, Hirofumi NAGAYAMA

**Affiliations:** 1 Kanagawa University of Human Services, Faculty of Health and Social Service, School of Rehabilitation, Division of Occupational Therapy Program, Yokosuka, Kanagawa; 2 Department of Rehabilitation, Yamagata Prefectural Central Hospital, Yamagata; 3 Department of Rehabilitation Therapy, Saiseikai Higashikanagawa Rehabilitation Hospital, Yokohama, Japan

**Keywords:** stroke, paresis, outcome assessment, patient discharge, rehabilitation, physical exercises

## Abstract

**Objective:**

To identify profiles of stroke patient benefitting from additional training, using latent class analysis.

**Design:**

Retrospective observational study.

**Patients:**

Patients with stroke (*n* = 6,875) admitted to 42 recovery rehabilitation units in Japan between January 2005 and March 2016 who were registered in the Japan Association of Rehabilitation Database.

**Methods:**

The main outcome measure was the difference in Functional Independence Measure (FIM) scores between admission and discharge (referred to as “gain”). The effect of additional training, categorized as usual care (no additional training), self-exercise, training with hospital staff, or both exercise (combining self-exercise and training with hospital staff), was assessed through multiple regression analyses of latent classes.

**Results:**

Applying inclusion and exclusion criteria, 1185 patients were classified into 7 latent classes based on their admission characteristics (class size *n* = 82 (7%) to *n* = 226 (19%)). Patients with class 2 characteristics (right hemiparesis and modified dependence in the motor-FIM and cognitive-FIM) had positive FIM gain with additional training (95% confidence interval (95% CI) 0.49–3.29; *p* < 0.01). One-way analysis of variance revealed that training with hospital staff (95% CI 0.07–16.94; *p* < 0.05) and both exercises (95% CI 5.38–15.13; *p* < 0.01) led to a significantly higher mean FIM gain than after usual care.

**Conclusion:**

Additional training in patients with stroke with right hemiparesis and modified dependence in activities of daily living was shown to improve activities of daily living. Training with hospital staff combined with self-exercise is a promising rehabilitation strategy for these patients.

LAY ABSTRACT

This study, conducted in Japan, aimed to identify the profiles of patients with stroke who would benefit from extra training. The records of 6875 patients with stroke who had been admitted to rehabilitation units between 2005 and 2016 were analysed and the change in patients’ abilities between admission to the unit and discharge was determined. Some patients received additional training, while others did not. The results showed that patients with certain characteristics, such as right-side weakness and difficulty with daily activities, improved with extra training. Specifically, a combination of training from hospital staff and self-exercise was most effective. In summary, the study found that additional training can help stroke patients, especially those with right-side weakness and those who find daily activities challenging. Combining training from hospital staff with self-exercise seems to be a promising approach for rehabilitation in these patients.

Cerebrovascular disease presents significant global health challenges. It is ranked as the second-highest cause of death and third-highest cause of disability (1). Rehabilitating patients with cerebrovascular diseases is vital for enhancing functionality and independence, emphasizing the critical importance of achieving positive outcomes in this field. Reportedly, patients with stroke who spend longer periods in bed are more likely to experience a decline in activities of daily living (ADL), motor function, and cognitive function (2–4). Furthermore, it has been observed that complications, such as infections, can also impact the outcome of rehabilitation (5, 6). Active rehabilitation improves the clinical outcomes of patients with stroke, including balance ability, trunk control, walking speed (7, 8), post-discharge daily activities (9), shorter hospitalization periods (10), and long-term improvements (11, 12). Additional training, such as self-exercise, contributes to a comprehensive approach, improves independence in ADL and increases the rate of returning home (13, 14).

Previous studies have investigated the effectiveness of additional training on specific patient characteristics and training types (13–18). These studies identified factors, such as ADL severity and cognitive function, which influence the effectiveness of additional training. However, unresolved issues persist in terms of patient profiles and the effectiveness of different types of additional training, such as self-exercise, training programmes with hospital staff, or a combination of both.

By identifying comprehensive patient characteristics, including physical function, functional capacity, intervention duration, and other relevant factors, this study aimed to investigate the profiles of patients and determine the effective types of additional training, using latent class analysis (LCA). LCA is a type of structural equation modelling method used to obtain a heterogeneous classification within a target population, based on observable and unobserved concepts (latent variables). This study used LCA to identify patient characteristics that warrant the introduction of additional training and determine its effective combinations.

## METHODS

A retrospective observational study was conducted using data from the Japan Association of Rehabilitation Databases (JARD)(https://square.umin.ac.jp/JARD/index.html). All data were de-identified and the need for informed consent was waived. This study was approved by the Institutional Review Boards of Kanagawa University of Human Services (number 5-22-14).

### Database

Data for a sample of volunteer patients who were admitted to the participating hospitals between January 2005 and March 2016 were retrospectively collected from the JARD The data covered patients with various diagnoses and stages, including those with stroke in internal medicine and rehabilitation wards. The information recorded in the database included the National Institutes of Health Stroke Scale (NIHSS) and Functional Independence Measure (FIM) scores, patient age, stroke type, severity, duration, and type of rehabilitation, among other variables. Data from 33,657 patients were analysed, and data for 6,875 stroke patients admitted to acute rehabilitation wards in 42 hospitals were extracted for this study.

### Participants

Inclusion criteria were: (*i*) patients with post-stroke conditions admitted to acute rehabilitation wards registered in JARD between 2005 and 2015; (*ii*) those aged ≥ 18 years; and (*iii*) those who received rehabilitation (physical therapy, occupational therapy).

Exclusion criteria were: (*i*) patients aged < 18 years, (*ii*) those who were unable to receive rehabilitation during hospitalization; (*iii*) those with a length of stay < 14 days or ≥ 180 days; (*iv*) those with a pre-stroke modified Rankin scale score ≤ 1; and (*v*) those who had died or were transferred or moved to another ward due to deterioration in health status.

LCA was conducted using data for patients with cerebrovascular diseases and the classes were categorized. Due to concerns about compromising the significance of the results by excluding patients with cognitive function decline, as it might not sufficiently reflect real-world data, it was decided not to consider cognitive impairment as an inclusion or exclusion criteria.

### Additional training

Additional training consisted of self-exercise, and training with hospital staff, extracted from the JARD items. The specifics and intensity of self-exercise varied, and comprehensive information was not available for all facilities. However, a previous study (13) indicated that therapists and nurses guided the planning of self-exercise and training with hospital staff, with a primary focus on activities such as standing up, transferring (bed/chair/wheelchair), and walking. Thus, the purpose of additional training was to complement compensatory rehabilitation by providing repetitive practice of specific activities and movements. Participants were categorized into 4 groups based on the implementation status of additional training: usual care (no additional training), self-exercise, training with hospital staff, or a combination of the last 2 groups.

### Outcomes

The primary outcome measure was the change in ADL performance assessed using the FIM score, which captured the difference between the FIM scores at discharge and admission. The FIM is a standardized assessment tool that evaluates the level of assistance required by individuals with disabilities to safely and efficiently perform basic ADLs. It comprises 18 items that assess both motor (13 items) and cognitive functions (5 items) (18, 19). Each item is rated on a scale of 1–7, ranging from total assistance to complete independence. The total score range is 18–126, with higher scores indicating better functional status (20, 21).

### Hospitalization outcome variables classified using latent class analysis

Age, affected side, functional ability, and cognitive ability were selected as hospitalization outcome variables classified using LCA based on previous research and clinical considerations. Sex data were extracted from the JARD items and classified into 2 categories: male = 0 and female = 1. Age data were extracted from the JARD items and classified into 5 categories: ≤ 54, 55–64, 65–74, 75–84, and ≥ 85 years. The affected side was classified into 4 categories based on the site of motor paralysis as a sequela of stroke: right, left, both, and none. Functional and cognitive abilities at admission were evaluated using FIM. Based on previous studies (22, 23), the FIM score was classified into 3 groups: complete dependence (1–2), modified dependence (3–5), and independence (6–7). The following items were selected as outcome variables for LCA: functional ability, such as eating, bowel control, transferring (bed/chair/wheelchair), transferring (toilet), transferring (bathing/showering), and locomotion (walking/wheelchair), and cognitive ability, such as comprehension, expression, social interaction, problem solving, and memory.

### Variable selection for multiple regression analysis

For each class classified by LCA, the dependent variable was set as the FIM score, and multiple regression analysis was performed. The independent variables were as follows: (*i*) implementation of additional training (0, usual care; 1, self-exercise; 2, training with hospital staff; 3, both); (*ii*) amount of rehabilitation per day (units/day); (*iii*) length of stay (days); (*iv*) implementation of conferences (1, regular; 2, regular with additional ad hoc sessions); and (*v*) provision of assistive devices (0, no; 1, yes). In addition, the covariates included variables that have been reported to affect ADL in patients with cerebrovascular diseases, such as age and FIM score at admission. The duration of daily rehabilitation was defined as 1 unit according to the guidelines of the Japanese health insurance, which corresponds to 20 min of rehabilitation (physical therapy, occupational therapy, and speech therapy).

### Post hoc analysis using one-way analysis of variance

If the multiple regression analysis confirmed that additional training had an impact on the FIM score, post hoc analysis was conducted using one-way analysis of variance (ANOVA) to examine which additional training contributed to the change in the FIM score.

### Statistical analysis

LCA was performed using the selected outcome variables to evaluate the patient’s condition at admission. As conducting a complete LCA with 13 variables violated the independence constraint of the model, the variable defining the most latent classes was selected using the swap-stepwise algorithm based on a Bayesian model comparison. The optimal number of latent classes in the latent model was determined based on the Bayesian information criterion (BIC), entropy, Vuong-Lo-Mendell-Rubin test, and combinations of class sizes. To handle missing data, the default option of Full Information Maximum Likelihood in Latent GOLD^®^ (version 6.043) was used. LCA was performed using Latent GOLD^®^ (version 6.0; Statistical Innovations, Arlington, MA, USA). For comparison of baseline characteristics among the participants, one-way ANOVA was used for continuous data and the χ^2^ test was used for categorical data.

Multiple regression analysis was used to examine the impact of additional training on FIM scores, excluding the influence of covariates. Variables with a variance inflation factor > 10.0 were excluded in order to avoid multicollinearity.

All analyses were conducted using Stata 15.1 (Stata Corp., College Station, TX, USA). Continuous data are presented as mean values and standard deviations (SD). Categorical data were assessed in terms of percentages (%). The significance level for multiple comparisons using continuous data was set at *p* < 0.01. χ^2^ tests for categorical data and multiple regression analyses were conducted at a significance level of *p* < 0.05.

## RESULTS

### Patient characteristics

**Fig. 1:**
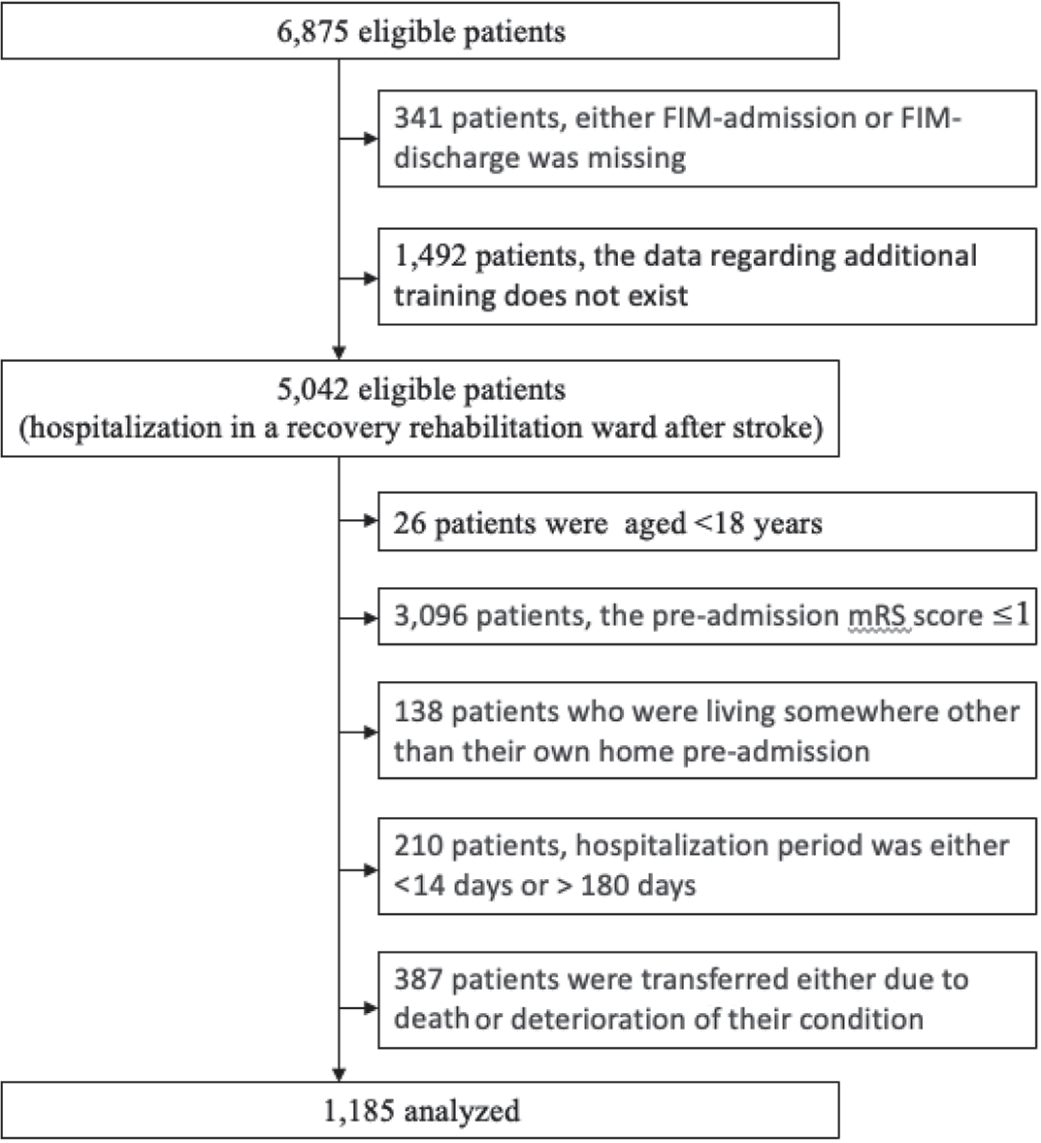
Flowchart of participant selection after applying the inclusion and exclusion criteria in this study. FIM: Functional Independence Measure; mRS: Modified Rankin scale.

After applying the inclusion and exclusion criteria, 1,185 patients were included in the study (Fig. 1). Patients’ baseline characteristics at admission are summarized in Table I. Mean age at admission for the overall sample was 67.3 (13.1) years and length of stay was 99.9 (42.5) days. Total FIM score, as well as the functional and cognitive aspect scores of the FIM, improved from admission to discharge.

**Table I:** Characteristics of study patients

Continuous variable, mean (SD)	Total		Usual care		Self-exercise		Hospital-staff training		Both exercise	
	N = 1185		N = 268		N = 107		N = 127		N = 683	
Age, years	67.3 (13.1)		71.6 (11.8)		65.1 (13.9)		72.2 (11.6)		65.0 (13.1)	
Length of stay, days	99.9 (42.5)		102.4 (45.9)		90.6 (39.3)		104.2 (40.9)		99.5 (41.7)	
Intervention by PT, OT and ST (units/day)	4.8 (1.9)		4.9 (1.8)		5.6 (1.7)		3.9 (1.6)		4.8 (1.9)	
	Admission	Discharge	Admission	Discharge	Admission	Discharge	Admission	Discharge	Admission	Discharge
Motor-FIM, Point	49.3 (22.7)	70.3 (19.6)	45.8 (23.8)	64.7 (23.7)	66.3 (18.9)	80.9 (11.1)	32.7 (20.0)	54.7 (22.6)	51.2 (20.9)	73.8 (15.7)
Cognition-FIM, point	23.5 (8.6)	27.4 (7.5)	21.4 (9.2)	24.4 (8.6)	27.0 (7.2)	29.7 (5.9)	16.3 (7.5)	20.8 (6.7)	25.2 (7.8)	29.4 (6.3)
FIM (total), point	72.6 (28.7)	97.3 (25.6)	67.1 (30.3)	88.9 (30.4)	92.1 (24.2)	109.9 (15.8)	49.2 (24.8)	75.5 (27.4)	76.0 (26.1)	102.6 (20.7)
Category variable, *n* (%)										
Sex, male, *n* (%)	460 (38.9)		109 (40.7)		33 (30.8)		39 (30.7)		279 (40.9)	
Age-group, *n* (%)										
≥ 54 years	183 (15.4)		21 (7.8)		19 (17.8)		9 (7.1)		134 (19.6)	
55–64 years	255 (21.5)		39 (14.6)		26 (24.3)		20 (15.7)		170 (24.9)	
65–74 years	339 (28.6)		79 (29.5)		34 (31.8)		33 (26.0)		193 (28.3)	
75–84 years	334 (28.2)		99 (36.9)		24 (22.4)		49 (38.6)		162 (23.7)	
≥ 85 years	72 (6.1)		30 (11.2)		4 (3.7)		16 (12.6)		22 (3.2)	
Missing	2 (0.2)		0 (0.0)		0 (0.0)		0 (0.0)		2 (0.3)	
Major stroke types, *n* (%										
Cerebral infarction	719 (60.7)		168 (62.7)		58 (54.2)		79 (62.2)		414 (60.6)	
Cerebral haemorrhage	375 (31.6)		82 (30.6)		35 (32.7)		35 (27.6)		223 (32.7)	
Subarachnoid haemorrhage	71 (6.0)		11 (4.1)		11 (10.3)		13 (10.2)		36 (5.3)	
Missing	20 (1.7)		7 (2.6)		3 (2.8)		0 (0.0)		10 (1.5)	
Body side with post-stroke paralysis, *n* (%)										
Right	536 (45.2)		130 (48.5)		50 (46.7)		48 (37.8)		308 (45.1)	
Left	444 (37.5)		112 (41.8)		44 (41.1)		53 (41.7)		235 (34.4)	
Both	51 (4.3)		7 (2.6)		2 (1.9)		14 (11.0)		28 (4.1)	
Nothing	83 (7.0)		18 (6.7)		9 (8.4)		9 (7.1)		47 (6.9)	
Missing	71 (6.0)		1 (0.4)		2 (1.9)		3 (2.4)		65 (9.5)	
Conducting conferences, *n* (%)										
Peliodic	660 (55.7)		193 (72.0)		73 (68.2)		39 (30.7)		355 (52.0)	
Periodic and as needed	508 (42.9)		71 (26.5)		31 (29.0)		87 (68.5)		319 (46.7)	
Missing	17 (1.4)		4 (1.5)		3 (2.8)		1 (0.8)		9 (1.3)	
Orthotic prescriptions, *n* (%)										
Yes	274 (23.1)		61 (22.8)		18 (16.8)		20 (15.7)		175 (25.6)	
No	639 (53.9)		206 (76.9)		89 (83.2)		47 (37.0)		297 (43.5)	
Missing	272 (23.0)		1 (0.4)		0 (0.0)		60 (47.2)		211 (30.9)	
FIM eating, *n* (%)										
Complete dependence (1, 2)	157 (13.2)	42 (3.5)	66 (24.6)	14 (5.2)	2 (1.9)	0 (0.0)	32 (25.2)	12 (9.4)	57 (8.3)	16 (2.3)
Modified dependence (3–5)	372 (31.4)	185 (15.6)	58 (21.6)	41 (15.3)	13 (12.1)	3 (2.8)	56 (44.1)	45 (35.4)	245 (35.9)	96 (14.1)
Independence (6, 7)	654 (55.2)	955 (80.6)	144 (53.7)	213 (79.5)	92 (86.0)	104 (97.2)	39 (30.7)	70 (55.1)	379 (55.5)	568 (83.2)
Missing	2 (0.2)	3 (0.3)	0 (0.0)	0 (0.0)	0 (0.0)	0 (0.0)	0 (0.0)	0 (0.0)	2 (0.3)	3 (0.4)
FIM toileting, *n* (%)										
Complete dependence (1, 2)	434 (36.6)	130 (11.0)	113 (42.2)	53 (19.8)	10 (9.3)	2 (1.9)	81 (63.8)	35 (27.6)	230 (33.7)	40 (5.9)
Modified dependence (3–5)	396 (33.4)	182 (15.4)	97 (36.2)	56 (20.9)	41 (38.3)	11 (10.3)	31 (24.4)	42 (33.1)	227 (33.2)	73 (10.7)
Independence (6, 7)	350 (29.5)	870 (73.4)	57 (21.3)	159 (59.3)	56 (52.3)	94 (87.9)	15 (11.8)	50 (39.4)	222 (32.5)	567 (83.0)
Missing	5 (0.4)	3 (0.3)	1 (0.4)	0 (0.0)	0 (0.0)	0 (0.0)	0 (0.0)	0 (0.0)	4 (0.6)	3 (0.4)
FIM transfer (bed/wheelchair), *n* (%)										
Complete dependence (1, 2)	265 (22.4)	64 (5.4)	78 (29.1)	23 (8.6)	8 (7.5)	1 (0.9)	63 (49.6)	22 (17.3)	116 (17.0)	18 (2.6)
Modified dependence (3–5)	424 (35.8)	168 (14.2)	100 (37.3)	61 (22.8)	34 (31.8)	6 (5.6)	39 (30.7)	34 (26.8)	251 (36.7)	67 (9.8)
Independence (6, 7)	363 (30.6)	869 (73.3)	63 (23.5)	162 (60.4)	53 (49.5)	95 (88.8)	13 (10.2)	51 (40.2)	234 (34.3)	561 (82.1)
Missing	133 (11.2)	84 (7.1)	27 (10.1)	22 (8.2)	12 (11.2)	5 (4.7)	12 (9.4)	20 (15.7)	82 (12.0)	37 (5.4)
FIM transfer (toilet), *n* (%)										
Complete dependence (1, 2)	308 (26.0)	72 (6.1)	94 (35.1)	29 (10.8)	8 (7.5)	1 (0.9)	68 (53.5)	23 (18.1)	138 (20.2)	19 (2.8)
Modified dependence (3–5)	531 (44.8)	229 (19.3)	116 (43.3)	76 (28.4)	46 (43.0)	11 (10.3)	46 (36.2)	46 (36.2)	323 (47.3)	96 (14.1)
Independence (6, 7)	342 (28.9)	880 (74.3)	57 (21.3)	161 (60.1)	52 (48.6)	94 (87.9)	13 (10.2)	58 (45.7)	220 (32.2)	567 (83.0)
Missing	4 (0.3)	4 (0.3)	1 (0.4)	2 (0.7)	1 (0.9)	1 (0.9)	0 (0.0)	0 (0.0)	2 (0.3)	1 (0.1)
FIM transfer (bath/shower), *n* (%)										
Complete dependence (1, 2)	596 (50.3)	217 (18.3)	143 (53.4)	74 (27.6)	21 (19.6)	5 (4.7)	95 (74.8)	39 (30.7)	337 (49.3)	99 (14.5)
Modified dependence (3–5)	485 (40.9)	559 (47.2)	111 (41.4)	128 (47.8)	70 (65.4)	56 (52.3)	29 (22.8)	71 (55.9)	275 (40.3)	304 (44.5)
Independence (6,7)	98 (8.3)	405 (34.2)	14 (5.2)	65 (24.3)	15 (14.0)	45 (42.1)	2 (1.6)	16 (12.6)	67 (9.8)	279 (40.8)
Missing	6 (0.5)	4 (0.3)	0 (0.0)	1 (0.4)	1 (0.9)	1 (0.9)	1 (0.8)	1 (0.8)	4 (0.6)	1 (0.1)
FIM locomotion (walk/wheelchair), *n* (%)										
Complete dependence (1, 2)	633 (53.4)	245 (20.7)	133 (49.6)	49 (18.3)	18 (16.8)	1 (0.9)	98 (77.2)	45 (35.4)	384 (56.2)	150 (22.0)
Modified dependence (3–5)	348 (29.4)	280 (23.6)	87 (32.5)	62 (23.1)	48 (44.9)	16 (15.0)	23 (18.1)	50 (39.4)	190 (27.8)	152 (22.3)
Independence (6,7)	199 (16.8)	654 (55.2)	48 (17.9)	155 (57.8)	39 (36.4)	89 (83.2)	6 (4.7)	32 (25.2)	106 (15.5)	378 (55.3)
Missing	5 (0.4)	6 (0.5)	0 (0.0)	2 (0.7)	2 (1.9)	1 (0.9)	0 (0.0)	0 (0.0)	3 (0.4)	3 (0.4)
FIM comprehension, *n* (%)										
Complete dependence (1,2)	163 (13.8)	72 (6.1)	65 (24.3)	37 (13.8)	9 (8.4)	4 (3.7)	37 (29.1)	12 (9.4)	52 (7.6)	19 (2.8)
Modified dependence (3–5)	478 (40.3)	338 (28.5)	100 (37.3)	88 (32.8)	31 (29.0)	23 (21.5)	75 (59.1)	85 (66.9)	272 (39.8)	142 (20.8)
Independence (6,7)	537 (45.3)	766 (64.6)	103 (38.4)	142 (53.0)	65 (60.7)	79 (73.8)	15 (11.8)	30 (23.6)	354 (51.8)	515 (75.4)
Missing	7 (0.6)	9 (0.8)	0 (0.0)	1 (0.4)	2 (1.9)	1 (0.9)	0 (0.0)	0 (0.0)	5 (0.7)	7 (1.0)
FIM expression, *n* (%)										
Complete dependence (1, 2)	209 (17.6)	102 (8.6)	60 (22.4)	35 (13.1)	9 (8.4)	4 (3.7)	46 (36.2)	22 (17.3)	94 (13.8)	41 (6.0)
Modified dependence (3–5)	438 (37.0)	326 (27.5)	109 (40.7)	102 (38.1)	31 (29.0)	22 (20.6)	57 (44.9)	69 (54.3)	241 (35.3)	133 (19.5)
Independence (6, 7)	530 (44.7)	748 (63.1)	98 (36.6)	130 (48.5)	65 (60.7)	80 (74.8)	24 (18.9)	36 (28.3)	343 (50.2)	502 (73.5)
Missing	8 (0.7)	9 (0.8)	1 (0.4)	1 (0.4)	2 (1.9)	1 (0.9)	0 (0.0)	0 (0.0)	5 (0.7)	7 (1.0)
FIM social interaction, *n* (%)										
Complete dependence (1, 2)	192 (16.2)	80 (6.8)	63 (23.5)	36 (13.4)	5 (4.7)	2 (1.9)	51 (40.2)	18 (14.2)	73 (10.7)	24 (3.5)
Modified dependence (3–5)	412 (34.8)	291 (24.6)	81 (30.2)	84 (31.3)	31 (29.0)	17 (15.9)	56 (44.1)	70 (55.1)	244 (35.7)	120 (17.6)
Independence (6, 7)	574 (48.4)	806 (68.0)	124 (46.3)	148 (55.2)	69 (64.5)	87 (81.3)	20 (15.7)	39 (30.7)	361 (52.9)	532 (77.9)
Missing	7 (0.6)	8 (0.7)	0 (0.0)	0 (0.0)	2 (1.9)	1 (0.9)	0 (0.0)	0 (0.0)	5 (0.7)	7 (1.0)
FIM problem solving, *n* (%)										
Complete dependence (1, 2)	291 (24.6)	161 (13.6)	98 (36.6)	68 (25.4)	9 (8.4)	3 (2.8)	64 (50.4)	42 (33.1)	120 (17.6)	48 (7.0)
Modified dependence (3–5)	536 (45.2)	475 (40.1)	102 (38.1)	110 (41.0)	52 (48.6)	43 (40.2)	61 (48.0)	72 (56.7)	321 (47.0)	250 (36.6)
Independence (6,7)	351 (29.6)	541 (45.7)	68 (25.4)	90 (33.6)	44 (41.1)	60 (56.1)	2 (1.6)	13 (10.2)	237 (34.7)	378 (55.3)
Missing	7 (0.6)	8 (0.7)	0 (0.0)	0 (0.0)	2 (1.9)	1 (0.9)	0 (0.0)	0 (0.0)	5 (0.7)	7 (1.0)
FIM memory, *n* (%)										
Complete dependence (1, 2)	239 (20.2)	118 (10.0)	82 (30.6)	60 (22.4)	7 (6.5)	4 (3.7)	50 (39.4)	23 (18.1)	100 (14.6)	31 (4.5)
Modified dependence (3–5)	501 (42.3)	386 (32.6)	105 (39.2)	94 (35.1)	44 (41.1)	28 (26.2)	70 (55.1)	83 (65.4)	282 (41.3)	181 (26.5)
Independence (6, 7)	438 (37.0)	673 (56.8)	81 (30.2)	114 (42.5)	54 (50.5)	74 (69.2)	7 (5.5)	21 (16.5)	296 (43.3)	464 (67.9)
Missing	7 (0.6)	8 (0.7)	0 (0.0)	0 (0.0)	2 (1.9)	1 (0.9)	0 (0.0)	0 (0.0)	5 (0.7)	7 (1.0)

Daily amount of rehabilitation was defined as 1 unit according to the guidelines of the Japanese health insurance, which corresponds to 20 min of rehabilitation (PT, OT, ST).

FIM: Functional Independence Measure (range 18–126; a higher score indicated higher independence); OT: occupational therapist; PT: physical therapist; ST: speech language therapist.

### Latent classes of patients’ characteristics at admission

LCA was performed on data from 1,185 patients, based on their admission status, to classify them into latent classes in 2 stages.

In the first stage, model fit statistics were used to select the optimal class from the latent class models with 1–14 classes (Table SI). Based on the difference in the BIC/Akaike information criterion (AIC) fit between the models, a plateau in BIC reduction was observed in the 8-class model. Table SII shows the class sizes and item response probabilities for the admission-related items within the 8-class model. For example, a class size of 20% for class 1 indicates that 20% of all patients belong to this class, and 90% of class 1 patients have an “independence” response for the item “FIM eating”. In addition, considering the item response probabilities for each item, items that did not significantly contribute to the class classification or had similar patterns were excluded. The item “sex” had similar response probabilities for “male” and “female” and was excluded from the analysis as it did not significantly impact class classification. Similarly, “FIM transfer (bed/chair/wheelchair)” was excluded since its response probabilities showed patterns similar to those of “FIM transfer (toilet)”, which was considered to be important in previous studies (20, 22). Similarly, “FIM transfer (bath/shower)” was excluded as its response probabilities showed patterns similar to those of “FIM locomotion”, which was emphasized in previous studies (22, 23). In addition, “FIM comprehension” was excluded as its response probabilities showed patterns similar to those of “FIM expression”, which is emphasized at discharge (22, 23). Similarly, “FIM memory” was excluded as its response probabilities showed patterns similar to those of “FIM social interaction” and “FIM problem solving”, which are important ADL items at discharge (22, 23).

In the second stage, model fit statistics were used to select the optimal class from the latent class models with 1–9 classes (Table SIII). Based on the difference in the BIC/AIC fit between the models, a plateau in BIC reduction was observed with the 7-class model. Table II presents the class sizes and item response probabilities for admission-related items within the 7-class model. The overall characteristics of each class indicated that those in class 1 had the mildest condition (independent of both motor and cognitive items) while those in class 5 had the most severe condition (complete dependence in both motor and cognitive items). The remaining classes exhibited different degrees of severity, and the patient characteristics were clinically acceptable. The baseline characteristics of the participants in each class are shown in Table SIV.

**Table II:** Class sizes (9 items)

	Class 1	Class 2	Class 3	Class 4	Class 5	Class 6	Class 7	Overall
Class size, *n* (%)	226 (19)	226 (19)	214 (18)	222 (18)	117 (10)	98 (8)	82 (7)	
Summary								
Age-group, years	≤ 85	65–84	65–84	55–84	65–84	55–84	65–84	
Body side with post-stroke paralysis	Right, left	Right	Left	Left	Right	Right	Right	
Functional ability	Independence	Modified dependence (eating: independence)	Complete dependence (eating: modified dependence)	Modified dependence (eating: independence)	Complete dependence	Independence	Modified dependence (locomotion: complete dependence)	
Cognitive ability	Independence	Modified dependence	Modified dependence	Independence	Complete dependence	Modified dependence	Complete dependence (comprehension: Modified dependence)	
Age-group, %								
≤ 54 years	**24%**	15%	13%	15%	7%	19%	7%	15%
55–64 years	**24%**	15%	18%	**33%**	18%	**28%**	10%	22%
65–74 years	**27%**	**31%**	**28%**	**25%**	**32%**	**26%**	**36%**	29%
75–84 years	**21%**	**28%**	**32%**	**24%**	**37%**	**25%**	**42%**	28%
≥ 85 years	4%	11%	10%	3%	6%	2%	5%	6%
Body side with post-stroke paralysis, %								
Right	**44%**	**56%**	38%	35%	**58%**	**59%**	**68%**	48%
Left	**42%**	34%	**52%**	**53%**	37%	17%	17%	40%
Both	1%	3%	8%	5%	4%	2%	9%	5%
Nothing	13%	6%	2%	6%	0%	22%	5%	7%
FIM eating, %								
Complete dependence (1, 2)	1%	2%	17%	6%	**71%**	2%	15%	13%
Modified dependence (3–5)	7%	35%	**61%**	26%	28%	12%	**56%**	31%
Independence (6, 7)	**92%**	**63%**	21%	**68%**	1%	**86%**	28%	55%
FIM toileting, %								
Complete dependence (1, 2)	0%	13%	**91%**	31%	**100%**	0%	30%	37%
Modified dependence (3–5)	6%	**78%**	9%	**60%**	0%	8%	**66%**	34%
Independence (6, 7)	**94%**	9%	0%	8%	0%	**92%**	4%	30%
FIM transfer (toilet), %								
Complete dependence (1, 2)	0%	0%	**70%**	19%	**99%**	0%	0%	26%
Modified dependence (3–5)	4%	**98%**	30%	**74%**	1%	2%	**94%**	45%
Independence (6, 7)	**96%**	2%	0%	7%	0%	**98%**	6%	29%
FIM locomotion(walk/wheelchair), %								
Complete dependence (1, 2)	20%	39%	**92%**	**59%**	**99%**	21%	**49%**	54%
Modified dependence (3–5)	24%	**60%**	7%	38%	1%	23%	**47%**	29%
Independence (6, 7)	**56%**	1%	1%	2%	0%	**57%**	3%	17%
FIM comprehension, %								
Complete dependence (1, 2)	0%	2%	9%	0%	**78%**	14%	43%	14%
Modified dependence (3–5)	8%	**68%**	**76%**	4%	22%	**63%**	**55%**	41%
Independence (6, 7)	**92%**	30%	15%	**96%**	1%	23%	2%	46%
FIM social interaction, %								
Complete dependence (1, 2)	0%	2%	11%	1%	**85%**	8%	**68%**	16%
Modified dependence (3–5)	2%	**67%**	**68%**	2%	10%	**73%**	23%	35%
Independence (6, 7)	**98%**	32%	20%	**97%**	5%	19%	9%	49%
FIM problem solving, %								
Complete dependence (1, 2)	1%	7%	29%	2%	**100%**	16%	**91%**	25%
Modified dependence (3–5)	21%	**92%**	**69%**	20%	0%	**84%**	8%	46%
Independence (6, 7)	**79%**	1%	2%	**78%**	0%	0%	1%	30%

FIM: Functional Independence Measure (range, 18–126, a higher score indicated higher independence). Bold text in the table indicates the stratum with the highest probability of affiliation.

### Effect of additional training on activities of daily living

Multiple regression analyses using the FIM score as the dependent variable were performed for each class classified using LCA. The independent variables included: (*i*) implementation status of additional training, (*ii*) amount of rehabilitation per day, (*iii*) length of stay, (*iv*) implementation of conferences, and (*v*) prescription of assistive devices. The selected covariates included age, admission FIM (motor and cognitive), and major stroke type. Table III presents the results of the multiple regression analyses for each class. The study findings demonstrated that additional training had a statistically significant positive effect on the FIM score of class 2 patients with cerebrovascular diseases (B = 1.89; 95% CI 0.49–3.29; *p* < 0.01). In addition, motor-FIM (admission), FIM problem solving, age, major stroke type, and length of stay were identified as predictive factors for the FIM score in each class.

**Table III:** Effect of additional training

	Class (*p*-value)	B	SE	95% CI	*p*-value
FIM score	Class 1 (*p* < 0.001)	–0.19	0.33	–0.85–0.46	0.563
	Class 2 (*p* < 0.001)	1.89	0.71	0.49–3.29	0.008*
	Class 3 (*p* < 0.001)	0.32	1.46	–2.57–3.21	0.827
	Class 4 (*p* < 0.001)	0.05	0.72	–1.36–1.47	0.942
	Class 5 (*p* < 0.001)	–1.95	2.06	–6.05–2.16	0.349
	Class 6 (*p* < 0.001)	0.85	0.71	–0.58–2.27	0.240
	Class 7 (*p* < 0.001)	0.07	1.71	–3.35–3.50	0.966
Motor-FIM score	Class 1 (*p* < 0.001)	–0.49	0.29	–1.06–0.08	0.093
	Class 2 (*p* < 0.001)	1.28	0.59	0.12–2.44	0.031*
	Class 3 (*p* < 0.001)	–0.68	1.19	–3.04–1.68	0.569
	Class 4 (*p* < 0.001)	–0.04	0.64	–1.30–1.22	0.947
	Class 5 (*p* < 0.001)	–1.39	1.68	–4.74–1.95	0.410
	Class 6 (*p* < 0.001)	–0.14	0.45	–1.05–0.76	0.756
	Class 7 (*p* = 0.32)	0.15	1.30	–2.45–2.75	0.908

Multiple regression analysis: adjusted for intervention by PT, OT and ST, length of stay, conducting conferences, orthotic prescriptions, age, major stroke types, Motor-FIM (admission), Cognition-FIM (admission). B: regression coefficient or estimated value; FIM: Functional Independence Measure; SE: standard error; 95% CI: 95% confidence interval.

Furthermore, ANOVA with the FIM score as the dependent variable was conducted for class 2 (Table IV). Additional training had a significant effect on FIM score (F [3, 225] = 9.28; *p* < 0.01). Post hoc tests using Tukey’s honest significant difference indicated that the mean score of hospital-staff training was significantly higher than that of usual care (95% CI 0.07–16.94; *p* < 0.05). In addition, both self-exercise and training with hospital staff had a significantly higher mean score compared with that of usual care (95% CI 5.38–15.13; *p* < 0.01).

**Table IV:** Comparison of each additional training method in class 2

				Mean difference	Standard error	95% CI	*p*-value
FIM score	Usual care	vs	Self-exercise	–5.12	3.26	–13.56 to 3.31	0.396
F (3, 225) = 9.28, *p* < 0.01	Usual care	vs	Hospital-staff training	–8.51	3.26	–16.94 to –0.07	0.047*
	Usual care	vs	Both exercise	–10.76	2.08	–16.13 to –5.38	0.000*
	Self-exercise	vs	Hospital-staff training	–3.38	3.87	–13.39 to 6.63	0.818
	Self-exercise	vs	Both exercise	–5.63	2.94	–13.24 to 1.98	0.224
	Hospital-staff training	vs	Both exercise	2.25	2.94	–5.36 to 9.86	0.870
Motor-FIM score	Usual care	vs	Self-exercise	–4.50	2.85	–11.87 to 2.88	0.393
F (3, 214) = 7,66, *p* < 0.01	Usual care	vs	Hospital-staff training	–8.30	2.80	–15.55 to –1.05	0.018*
	Usual care	vs	Both exercise	–8.42	1.81	–13.10 to –3.73	0.000*
	Self-exercise	vs	Hospital-staff training	–3.80	3.35	–12.49 to 4.88	0.669
	Self-exercise	vs	Both exercise	–3.92	2.59	–10.62 to 2.77	0.429
	Hospital-staff training	vs	Both exercise	–0.12	2.53	–6.68 to 6.44	1.000

Additional training, categorized as usual care (no additional training), self-exercise, training with hospital staff, or both exercise (combining self-exercise and training with hospital staff).

FIM: Functional Independence Measure (range, 18–126, a higher score indicated higher independence).

## DISCUSSION

In this study, patients with cerebrovascular disease were classified based on their admission status using LCA. Furthermore, specific patient profiles were identified, and the effects of additional training were evaluated. The results of the LCA classified the patients with cerebrovascular diseases into 7 classes. Accordingly, the study demonstrated that additional training appears to be effective for a subgroup of patients with the following characteristics: (*i*) age between 65 and 84 years, (*ii*) right hemiplegia, and (*iii*) modified dependence in the Motor-FIM (admission) and cognitive-FIM (admission).

Previous studies have investigated the effects of post-stroke rehabilitation interventions on social participation training and service utilization. However, reports on additional training remain limited and represent an ongoing area of research (24). Regarding additional training interventions, some studies have reported the effectiveness of repetitive practice of specific activities and movements, such as standing up, transferring, and walking (16), as well as the introduction of a nurse-led exercise intervention programme on basic motor function (Motor Assessment Scale) and ADL (Modified Barthel Index and FIM) in patients with cerebrovascular diseases (17, 18). Moreover, the effectiveness of training for language impairments has also been reported (25–27). Language impairments represent a significant factor that could impact both the mood of patients and the continuity of rehabilitation. Similarly, this study demonstrated the effectiveness of additional training in improving ADL. This study adds to the existing knowledge by revealing detailed patient characteristics that are responsive to additional training and effective training components.

The results of this study have 2 important implications. They clarify the patient characteristics for which additional training is indicated. Previous studies on patient characteristics have reported the effectiveness of additional training for patients with a total FIM score of < 56 (16). However, these studies did not reveal detailed patient characteristics. Previous studies examining the effects of additional training on functional improvement in patients with cerebrovascular diseases (28, 29) required the absence of cognitive impairment as an inclusion criterion; however, the effectiveness of additional training in patients with cognitive impairment remained unclear. This study revealed that additional training is effective for patients with right hemiplegia and modified dependence on Motor-FIM (admission) and cognitive-FIM (admission), providing a more detailed understanding of patient characteristics. Furthermore, this study clarified patient characteristics that were not influenced by additional training in terms of FIM gain.

The second implication is that a combination of additional training is effective in improving patients’ ADL. Previous studies on the content of additional training have reported the effectiveness of therapist-led self-exercise (15) and the introduction of a nurse-led exercise intervention programme (17, 18). However, it was unclear which type of exercise was more effective and the combined effects of these additional training approaches remained ambiguous. This study revealed that adding self-exercise to training with hospital staff has an additional effect in terms of FIM gain.

Previously, determining which patients should receive additional training and what type of additional training should be provided was dependent on the experience level of healthcare professionals. Therefore, the findings obtained in this study are expected to contribute to evidence-based selection of patient characteristics that warrant the introduction of additional training and effective combinations of additional training.

However, it is evident that the effectiveness of additional training for patients belonging to the class achieving independence in functional ability (class 1 and class 6) is limited owing to the ceiling effect of the Motor-FIM (30). In addition, patients belonging to a class with complete dependence on functional ability (class 3 and class 5) may be in a state in which sufficient or necessary additional training cannot be provided. Furthermore, the primary difference between participants in class 2 and class 4 is the side of body affected by post-stroke paralysis. Patients with left hemiplegia are more likely to exhibit spatial neglect than those with right hemiplegia (31), which has been reported to have a negative impact on motor paralysis and ADL independence (32). Disregarding spatial neglect can lead to limitations in ADL and is associated with poor functional recovery (33). While visual scanning training and mental imagery training are beneficial interventions for spatial neglect, their implementation requires specialized knowledge in spatial rehabilitation (34). Therefore, additional training for stroke patients with spatial neglect needs to be carefully planned and introduced. Consequently, patients with cerebrovascular diseases classified in a class other than Class 2 may require support in addition to repetitive practice. Furthermore, when evaluating the effectiveness of additional training, it is important to consider outcomes such as the quality of life of patients and caregivers, reduction in incidents such as falls, and maintenance of antigravity posture duration.

### Study limitations

This study has several limitations. First, there was a bias in the implementation of additional training, resulting in baseline differences between certain groups (e.g. classes 3, 5, and 7 had fewer instances of self-exercise, while classes 1 and 4 had fewer instances of training with hospital staff). However, these covariates were adjusted for in the final analyses. Secondly, detailed information on the specific content and dosage (frequency, amount, and duration) of the additional training provided is lacking. Moreover, various types of additional training, such as upper limb motor paralysis exercises (28, 35) and swallowing training (36), are typically provided to patients. Evaluating the effects of these diverse training types based solely on the FIM scores is challenging. Therefore, the results of this study cannot be generalized to all types of additional training. Finally, the database utilized in this study did not contain information regarding the localization of strokes. Future research should incorporate alternative outcome measures beyond ADL. It should also include comprehensive information on the content and dosage of additional training. This will aid in identifying effective and tailored training programmes based on patient characteristics.

### Conclusion

This study provides evidence supporting the effectiveness of additional training that targets ADL in patients with cerebrovascular disease who have right hemiplegia and modified ADL dependence. This will have the potential to guide decision-making processes regarding the introduction of additional training and avoiding subjective experiences through providing evidence-based insights based on comprehensive patient characteristics and multiple patient outcomes at admission. Furthermore, a combination of training with hospital staff and self-exercise is a promising rehabilitation approach. These findings emphasize the importance of personalized rehabilitation strategies. Further investigation is needed to refine and optimize the provision of additional training programmes for patients with cerebrovascular disease.

## Supplementary Material

IDENTIFYING PROFILES OF STROKE PATIENTS BENEFITTING FROM ADDITIONAL TRAINING: A LATENT CLASS ANALYSIS APPROACH
