# Supplementary material for: IDENTIFYING PROFILES OF STROKE PATIENTS BENEFITTING FROM ADDITIONAL TRAINING: A LATENT CLASS ANALYSIS APPROACH
Source: J Rehabil Med. 2024 Feb 21;56:22141. doi: 10.2340/jrm.v56.22141 (PMC10964030; doi:10.2340/jrm.v56.22141)
Supplement: IDENTIFYING PROFILES OF STROKE PATIENTS BENEFITTING FROM ADDITIONAL TRAINING: A LATENT CLASS ANALYSIS APPROACH [file JRM-56-22141-s1.pdf]

Supplementary material has been published as submitted. It has not been copyedited, typeset or checked for scientific content by Journal of Rehabilitation Medicine

Table SI. Model fit statistics (Classes 1–14)

	LL	BIC	ΔBIC	AIC	ΔAIC	df	VLMR	Entropy R2
1-Cluster	-16908.55	34029.42	—	33877.10	—	1155.00	—	1.00
2-Cluster	-14578.96	29589.65	4439.77	29279.93	4597.17	1124.00	4659.17	0.90
3-Cluster	-13642.76	27936.65	1653.00	27469.52	1810.40	1093.00	1872.40	0.93
4-Cluster	-13066.62	27003.76	932.89	26379.23	1090.29	1062.00	1152.29	0.94
5-Cluster	-12730.43	26550.79	452.97	25768.86	610.37	1031.00	672.37	0.93
6-Cluster	-12475.91	26261.15	289.64	25321.82	447.04	1000.00	509.04	0.93
7-Cluster	-12286.42	26101.57	159.58	25004.83	316.98	969.00	378.98	0.93
8-Cluster	-12139.22	26026.59	74.99	24772.44	232.39	938.00	294.39	0.92
9-Cluster	-12075.25	26118.05	-91.46	24706.50	65.94	907.00	127.94	0.92
10-Cluster	-12012.72	26212.38	-94.33	24643.43	63.07	876.00	125.07	0.92
11-Cluster	-11957.36	26321.06	-108.68	24594.71	48.72	845.00	110.72	0.91
12-Cluster	-11923.10	26471.95	-150.89	24588.20	6.52	814.00	68.52	0.90
13-Cluster	-11886.58	26618.32	-146.37	24577.17	11.03	783.00	73.03	0.91
14-Cluster	-11848.12	26760.79	-142.47	24562.24	14.93	752.00	76.93	0.91

Comparison of LCA models with different latent classes based on model selection statistics. Abbreviations: AIC, Akaike information criterion (lower values imply better model fit); BIC, Bayesian information criterion (lower values imply better model fit); df, degree of freedom; LCA, Latent class analysis; LL, Log-likelihood; VLMR, Vuong-Lo-Mendell-Rubin likelihood ratio test; Entropy, higher values imply better classification quality.

Table SII. Class sizes (14 items)

	Cluster1	Cluster2	Cluster3	Cluster4	Cluster5	Cluster6	Cluster7	Cluster8	Overall
Cluster Size	20%	18%	14%	13%	11%	10%	9%	5%	
Indicators									
**Sex**									
Male	62%	59%	62%	59%	67%	67%	54%	59%	61%
Female	39%	41%	38%	41%	33%	33%	46%	41%	39%
**Age-group**									
54≧	26%	15%	18%	11%	17%	8%	6%	10%	15%
55-64	26%	15%	29%	15%	26%	12%	20%	34%	22%
65-74	26%	31%	28%	30%	27%	31%	31%	26%	29%
75-84	19%	29%	23%	33%	24%	43%	37%	27%	28%
≧85	3%	10%	2%	11%	6%	5%	7%	2%	6%
**Body side with post-stroke paralysis**									
right	44%	55%	36%	35%	58%	66%	61%	27%	48%
left	43%	38%	53%	55%	18%	21%	34%	56%	40%
both	2%	4%	4%	7%	2%	8%	4%	9%	5%
nothing	10%	4%	7%	2%	22%	6%	0%	9%	7%
**FIM eating**									
complete Dependence(1,2)	2%	4%	2%	23%	2%	12%	73%	17%	13%
Modified Dependence(3-5)	8%	43%	22%	60%	11%	56%	26%	38%	31%
Independence(6,7)	90%	53%	76%	17%	87%	32%	2%	45%	55%
**FIM toileting**									
complete Dependence(1,2)	1%	31%	9%	93%	0%	40%	100%	89%	37%
Modified Dependence(3-5)	9%	64%	86%	7%	10%	55%	0%	10%	34%
Independence(6,7)	90%	5%	6%	0%	90%	4%	0%	1%	30%
**FIM transfer(bed/chair/wheelchair)**									
complete Dependence(1,2)	0%	1%	0%	84%	0%	0%	96%	56%	23%
Modified Dependence(3-5)	0%	97%	94%	16%	1%	90%	4%	44%	45%
Independence(6,7)	100%	3%	6%	0%	99%	10%	0%	0%	33%
**FIM transfer(toilet)**									
complete Dependence(1,2)	0%	1%	0%	98%	0%	3%	100%	72%	26%
Modified Dependence(3-5)	7%	99%	99%	2%	5%	93%	0%	28%	45%
Independence(6,7)	92%	0%	1%	0%	95%	4%	0%	0%	29%
**FIM transfer(bath/shower)**									
complete Dependence(1,2)	12%	52%	36%	99%	9%	54%	99%	100%	51%
Modified Dependence(3-5)	62%	48%	63%	1%	63%	46%	1%	0%	41%
Independence(6,7)	26%	0%	1%	0%	28%	0%	0%	0%	8%
**FIM locomotion(walk/wheelchair)**									
complete Dependence(1,2)	23%	51%	47%	94%	20%	53%	99%	91%	54%
Modified Dependence(3-5)	27%	48%	49%	5%	26%	45%	1%	9%	29%
Independence(6,7)	50%	1%	4%	1%	54%	3%	0%	0%	17%
**FIM comprehension**									
complete Dependence(1,2)	0%	2%	0%	10%	11%	35%	83%	0%	14%
Modified Dependence(3-5)	3%	78%	6%	80%	64%	59%	17%	4%	41%
Independence(6,7)	97%	21%	94%	10%	25%	6%	0%	96%	46%
**FIM expression**									
complete Dependence(1,2)	1%	6%	0%	20%	18%	44%	84%	0%	18%
Modified Dependence(3-5)	7%	67%	8%	66%	60%	51%	15%	18%	37%
Independence(6,7)	93%	27%	92%	14%	21%	5%	1%	82%	45%
**FIM social interaction**									
complete Dependence(1,2)	0%	2%	1%	18%	7%	50%	84%	2%	16%
Modified Dependence(3-5)	3%	66%	13%	67%	61%	42%	9%	6%	35%
Independence(6,7)	97%	32%	86%	15%	32%	8%	7%	93%	49%
**FIM problem solving**									
complete Dependence(1,2)	1%	2%	2%	35%	13%	85%	100%	5%	25%
Modified Dependence(3-5)	16%	98%	37%	64%	82%	14%	0%	17%	46%
Independence(6,7)	83%	0%	61%	1%	5%	1%	0%	78%	30%
**FIM memory**									
complete Dependence(1,2)	0%	5%	1%	23%	12%	61%	98%	0%	20%
Modified Dependence(3-5)	8%	90%	18%	74%	73%	38%	2%	15%	43%
Independence(6,7)	92%	5%	81%	3%	16%	2%	0%	85%	37%

Abbreviations: FIM; Functional Independence Measure

Table SIII. Model fit statistics (Classes 1–9)

	LL	BIC	ΔBIC	AIC	ΔAIC	df	VLMR	Entropy R2
1-Class	-11439,51	23027,65	—	22921,03	—	1164,00	—	1,00
2-Class	-10155,90	20616,13	2411,52	20397,80	2523,23	1142,00	2567,23	0,84
3-Class	-9692,19	19844,42	771,71	19514,38	883,42	1120,00	927,42	0,88
4-Class	-9432,09	19479,92	364,50	19038,18	476,20	1098,00	520,20	0,89
5-Class	-9288,34	19348,12	131,80	18794,67	243,51	1076,00	287,51	0,88
6-Class	-9153,48	19234,11	114,01	18568,96	225,72	1054,00	269,72	0,87
7-Class	-9058,15	19199,16	34,95	18422,30	146,65	1032,00	190,65	0,87
8-Class	-8989,73	19218,01	-18,86	18329,45	92,85	1010,00	136,85	0,87
9-Class	-8966,28	19326,82	-108,81	18326,55	2,90	988,00	46,90	0,87

Based on model selection statistics, comparison of LCA models with different latent classes. Abbreviations: AIC, Akaike information criterion (lower values imply better model fit); BIC, Bayesian information criterion (lower values imply better model fit); df, degree of freedom; LCA, Latent class analysis; LL, Log-likelihood; VLMR, Vuong-Lo-Mendell-Rubin likelihood ratio test; Entropy, higher values imply better classification quality

Table SIV. Characteristics of patients in all classes

Continuous variable, Mean(SD)	Class1 (N=226) Usual care N=42		Self exercise N=41		Hospital-staff training N=3		Both N=140			p-value
**Age**	69.6 (12.4)		61.1 (14.1)		67.0 (17.1)		62.2 (13.4)			0.010
**Length of stay**, days	67.2 (44.9)		68.5 (32.1)		38.3 (6.0)		74.4 (37.8)			0.278
Intervention by PT, OT and ST(units/day)	4.8 (1.8)		5.5 (1.7)		5.0 (1.8)		4.8 (2.0)			0.295
	**admission**	**discharge**	**admission**	**discharge**	**admission**	**discharge**	**admission**	**discharge**		
**Motor-FIM**(admission)	79.1 (9.6)	85.8 (5.3)	80.9 (7.9)	87.0 (3.9)	85.0 (8.5)	88.0 (4.2)	74.9 (9.0)	83.8 (5.8)	*	0.000
**Cognitive-FIM**(admission)	31.5 (3.3)	32.1 (3.0)	32.1 (3.2)	32.9 (3.0)	29.0 (4.0)	30.0 (3.6)	32.3 (3.0)	33.5 (2.1)		0.188
**FIM(total)**(admission)	110.1 (10.3)	117.1 (8.3)	112.0 (9.6)	119.9 (5.6)	107.0 (11.8)	110.3 (12.0)	107.0 (9.8)	117.1 (6.8)		0.024
**Category variable, N(%)**										
**Sex** male	17 (40.5%)		16 (39.0%)		2 (66.7%)		47 (33.6%)			0.553
**Age-group**										
≧54	5 (11.9%)		11 (26.8%)		1 (33.3%)		38 (27.1%)			0.316
55-64	9 (21.4%)		10 (24.4%)		0 (0.0%)		33 (23.6%)			
65-74	10 (23.8%)		14 (34.1%)		1 (33.3%)		40 (28.6%)			
75-84	15 (35.7%)		5 (12.2%)		1 (33.3%)		25 (17.9%)			
≧85	3 (7.1%)		1 (2.4%)		0 (0.0%)		4 (2.9%)			
Missing	0(0%)		0(0%)		0(0%)		0(0%)			
**Major Stroke Types**										
cerebral infarction	29 (69.0%)		27 (65.9%)		2 (66.7%)		100 (71.4%)			0.303
cerebral hemorrhage	8 (19.0%)		10 (24.4%)		0 (0.0%)		31 (22.1%)			
subarachnoid hemorrhage	1 (2.4%)		2 (4.9%)		1 (33.3%)		7 (5.0%)			
Missing	4 (9.5%)		2 (4.9%)		0 (0.0%)		2 (1.4%)			
**Body side with post-stroke paralysis**										
right	17 (40.5%)		19 (46.3%)		1 (33.3%)		58 (41.4%)			0.774
left	19 (45.2%)		19 (46.3%)		2 (66.7%)		52 (37.1%)			
both	0 (0.0%)		1 (2.4%)		0 (0.0%)		1 (0.7%)			
nothing	6 (14.3%)		2 (4.9%)		0 (0.0%)		20 (14.3%)			
Missing	0 (0.0%)		0 (0.0%)		0 (0.0%)		9 (6.4%)			
**Conducting conferences**										
peliodic	32 (76.2%)		26 (63.4%)		0 (0.0%)		72 (51.4%)		*	0.003
peliodic and as needed	9 (21.4%)		14 (34.1%)		3 (100.0%)		67 (47.9%)			
Missing	1 (2.4%)		1 (2.4%)		0 (0.0%)		1 (0.7%)			
**Orthotic prescriptions**										
Yes	2 (4.8%)		3 (7.3%)		0 (0.0%)		22 (15.7%)		*	0.027
No	39 (92.9%)		38 (92.7%)		2 (66.7%)		80 (57.1%)			
Missing	1 (2.4%)		0 (0.0%)		1 (33.3%)		38 (27.1%)			
	**admission**	**discharge**	**admission**	**discharge**	**admission**	**discharge**	**admission**	**discharge**		
**FIM eating**										
complete Dependence(1,2)	1 (2.4%)	0 (0.0%)	0 (0.0%)	0 (0.0%)	0 (0.0%)	0 (0.0%)	1 (0.7%)	0 (0.0%)		0.172
Modified Dependence(3-5)	0 (0.0%)	0 (0.0%)	1 (2.4%)	0 (0.0%)	0 (0.0%)	0 (0.0%)	15 (10.7%)	7 (5.0%)		
Independence(6,7)	41 (97.6%)	42 (100.0%)	40 (97.6%)	41 (100.0%)	3 (100.0%)	3 (100.0%)	124 (88.6%)	133 (95.0%)		
Missing	0 (0.0%)	0 (0.0%)	0 (0.0%)	0 (0.0%)	0 (0.0%)	0 (0.0%)	0 (0.0%)	0 (0.0%)		
**FIM toileting**										
complete Dependence(1,2)	0 (0.0%)	0 (0.0%)	0 (0.0%)	0 (0.0%)	0 (0.0%)	0 (0.0%)	0 (0.0%)	0 (0.0%)		0.908
Modified Dependence(3-5)	2 (4.8%)	0 (0.0%)	3 (7.3%)	0 (0.0%)	0 (0.0%)	0 (0.0%)	7 (5.0%)	0 (0.0%)		
Independence(6,7)	40 (95.2%)	42 (100.0%)	38 (92.7%)	41 (100.0%)	3 (100.0%)	3 (100.0%)	133 (95.0%)	140 (100.0%)		
Missing	0 (0.0%)	0 (0.0%)	0 (0.0%)	0 (0.0%)	0 (0.0%)	0 (0.0%)	0 (0.0%)	0 (0.0%)		
**FIM transfer(toilet)**										
complete Dependence(1,2)	0(0%)	0 (0.0%)	0(0%)	0 (0.0%)	0 (0.0%)	0 (0.0%)	0 (0.0%)	0 (0.0%)		0.805
Modified Dependence(3-5)	1 (2.4%)	1 (2.4%)	0 (0.0%)	0 (0.0%)	0 (0.0%)	0 (0.0%)	2 (1.4%)	1 (0.7%)		
Independence(6,7)	40 (95.2%)	40 (95.2%)	41 (100.0%)	41 (100.0%)	3 (100.0%)	3 (100.0%)	138 (98.6%)	139 (99.3%)		
Missing	1 (2.4%)	1 (2.4%)	0 (0.0%)	0 (0.0%)	0 (0.0%)	0 (0.0%)	0 (0.0%)	0 (0.0%)		
**FIM locomotion(walk/wheelchair)**										
complete Dependence(1,2)	2 (4.8%)	0 (0.0%)	2 (4.9%)	0 (0.0%)	1 (33.3%)	1 (33.3%)	38 (27.1%)	31 (22.1%)	*	0.005
Modified Dependence(3-5)	10 (23.8%)	1 (2.4%)	10 (24.4%)	2 (4.9%)	1 (33.3%)	0 (0.0%)	33 (23.6%)	6 (4.3%)		
Independence(6,7)	30 (71.4%)	40 (95.2%)	29 (70.7%)	39 (95.1%)	1 (33.3%)	2 (66.7%)	69 (49.3%)	103 (73.6%)		
Missing	0 (0.0%)	1 (2.4%)	0 (0.0%)	0 (0.0%)	0 (0.0%)	0 (0.0%)	0 (0.0%)	0 (0.0%)		
**FIM comprehension**										
complete Dependence(1,2)	0 (0.0%)	0 (0.0%)	0 (0.0%)	0 (0.0%)	0 (0.0%)	0 (0.0%)	0 (0.0%)	0 (0.0%)	*	0.001
Modified Dependence(3-5)	5 (11.9%)	3 (7.1%)	3 (7.3%)	2 (4.9%)	2 (66.7%)	2 (66.7%)	7 (5.0%)	3 (2.1%)		
Independence(6,7)	37 (88.1%)	39 (92.9%)	37 (90.2%)	39 (95.1%)	1 (33.3%)	1 (33.3%)	133 (95.0%)	137 (97.9%)		
Missing	0 (0.0%)	0 (0.0%)	1 (2.4%)	0 (0.0%)	0 (0.0%)	0 (0.0%)	0 (0.0%)	0 (0.0%)		
**FIM social interaction**										
complete Dependence(1,2)	0(0%)	0 (0.0%)	0 (0.0%)	0 (0.0%)	0 (0.0%)	0 (0.0%)	0 (0.0%)	0 (0.0%)		0.808
Modified Dependence(3-5)	1 (2.4%)	0 (0.0%)	0 (0.0%)	0 (0.0%)	0 (0.0%)	0 (0.0%)	3 (2.1%)	2 (1.4%)		
Independence(6,7)	41 (97.6%)	42 (100.0%)	40 (97.6%)	41 (100.0%)	3 (100.0%)	3 (100.0%)	137 (97.9%)	138 (98.6%)		
Missing	0 (0.0%)	0 (0.0%)	1 (2.4%)	0 (0.0%)	0 (0.0%)	0 (0.0%)	0 (0.0%)	0 (0.0%)		
**FIM problem solving**										
complete Dependence(1,2)	0 (0.0%)	0 (0.0%)	0 (0.0%)	0 (0.0%)	0 (0.0%)	0 (0.0%)	1 (0.7%)	0 (0.0%)	*	0.024
Modified Dependence(3-5)	12 (28.6%)	12 (28.6%)	8 (19.5%)	8 (19.5%)	3 (100.0%)	2 (66.7%)	24 (17.1%)	13 (9.3%)		
Independence(6,7)	30 (71.4%)	30 (71.4%)	32 (78.0%)	33 (80.5%)	0 (0.0%)	1 (33.3%)	115 (82.1%)	127 (90.7%)		
Missing	0 (0.0%)	0 (0.0%)	1 (2.4%)	0 (0.0%)	0 (0.0%)	0 (0.0%)	0 (0.0%)	0 (0.0%)		

Abbreviations: FIM; Functional Independence Measure (range, 18–126, a higher score indicated higher independence), OT; Occupational therapist, PT; Physical therapist, ST; Speech Language therapist

Continuous variable, Mean(SD)	Class2 (N=226)									
	Usual care		Self exercise		Hospital-staff training		Both			p-value
	N=50		N=21		N=21		N=134			
**Age**	74.3 (10.3)		71.0 (11.9)		76.4 (10.1)		67.0 (14.1)		*	0.001
**Length of stay**, days	100.1 (34.4)		108.5 (29.8)		93.2 (40.2)		97.9 (38.8)			0.568
Intervention by PT, OT and ST(units/day)	5.4 (2.0)		5.4 (1.7)		4.0 (1.4)		4.9 (1.8)			0.015
	**admission**	**discharge**	**admission**	**discharge**	**admission**	**discharge**	**admission**	**discharge**		
**Motor-FIM**(admission)	54.8 (10.0)	71.4 (14.2)	55.3 (8.7)	75.8 (9.9)	44.9 (9.5)	69.9 (9.6)	51.9 (10.3)	77.0 (7.9)	*	0.001
**Cognitive-FIM**(admission)	22.3 (4.7)	24.9 (5.5)	23.5 (4.5)	27.3 (5.5)	21.3 (3.6)	24.2 (4.3)	23.5 (3.7)	29.0 (3.9)		0.050
**FIM(total)**(admission)	77.1 (12.2)	96.5 (17.9)	77.2 (10.2)	101.8 (12.8)	66.2 (9.6)	94.1 (11.2)	74.7 (12.4)	104.9 (11.3)	*	0.004
**Category variable, N(%)**										
**Sex** male	20 (40.0%)		4 (19.0%)		5 (23.8%)		58 (43.6%)			0.080
**Age-group**										
≧54	2 (4.0%)		2 (9.5%)		0 (0.0%)		29 (21.6%)		*	0.026
55-64	6 (12.0%)		4 (19.0%)		3 (14.3%)		19 (14.2%)			
65-74	15 (30.0%)		6 (28.6%)		6 (28.6%)		44 (32.8%)			
75-84	18 (36.0%)		7 (33.3%)		6 (28.6%)		30 (22.4%)			
≧85	9 (18.0%)		2 (9.5%)		6 (28.6%)		11 (8.2%)			
Missing	0 (0.0%)		0 (0.0%)		0 (0.0%)		1 (0.7%)			
**Major Stroke Types**										
cerebral infarction	29 (58.0%)		8 (38.1%)		17 (81.0%)		84 (62.7%)		*	0.045
cerebral hemorrhage	20 (40.0%)		10 (47.6%)		2 (9.5%)		39 (29.1%)			
subarachnoid hemorrhage	0 (0.0%)		2 (9.5%)		2 (9.5%)		8 (6.0%)			
Missing	1 (2.0%)		1 (4.8%)		0 (0.0%)		3 (2.2%)			
**Body side with post-stroke paralysis**										
right	33 (66.0%)		12 (57.1%)		9 (42.9%)		69 (51.5%)			0.679
left	13 (26.0%)		7 (33.3%)		8 (38.1%)		39 (29.1%)			
both	1 (2.0%)		0 (0.0%)		2 (9.5%)		4 (3.0%)			
nothing	3 (6.0%)		2 (9.5%)		2 (9.5%)		6 (4.5%)			
Missing	0 (0.0%)		0 (0.0%)		0 (0.0%)		16 (11.9%)			
**Conducting conferences**										
peliodic	32 (64.0%)		17 (81.0%)		13 (61.9%)		69 (51.5%)			0.073
peliodic and as needed	17 (34.0%)		4 (19.0%)		8 (38.1%)		61 (45.5%)			
Missing	1 (2.0%)		0 (0.0%)		0 (0.0%)		4 (3.0%)			
**Orthotic prescriptions**										
Yes	9 (18.0%)		4 (19.0%)		3 (14.3%)		40 (29.9%)			0.012
No	41 (82.0%)		17 (81.0%)		13 (61.9%)		58 (43.3%)			
Missing	0 (0.0%)		0 (0.0%)		5 (23.8%)		36 (26.9%)			
	**admission**	**discharge**	**admission**	**discharge**	**admission**	**discharge**	**admission**	**discharge**		
**FIM eating**										
complete Dependence(1,2)	0 (0.0%)	0 (0.0%)	0 (0.0%)	0 (0.0%)	2 (9.5%)	2 (9.5%)	4 (3.0%)	1 (0.7%)	*	0.001
Modified Dependence(3-5)	7 (14.0%)	2 (4.0%)	4 (19.0%)	1 (4.8%)	10 (47.6%)	2 (9.5%)	55 (41.0%)	11 (8.2%)		
Independence(6,7)	43 (86.0%)	48 (96.0%)	17 (81.0%)	20 (95.2%)	9 (42.9%)	17 (81.0%)	74 (55.2%)	121 (90.3%)		
Missing	0 (0.0%)	0 (0.0%)	0 (0.0%)	0 (0.0%)	0 (0.0%)	0 (0.0%)	1 (0.7%)	1 (0.7%)		
**FIM toileting**										
complete Dependence(1,2)	5 (10.0%)	2 (4.0%)	0 (0.0%)	0 (0.0%)	3 (14.3%)	0 (0.0%)	17 (12.7%)	0 (0.0%)		0.478
Modified Dependence(3-5)	43 (86.0%)	13 (26.0%)	18 (85.7%)	4 (19.0%)	16 (76.2%)	10 (47.6%)	103 (76.9%)	7 (5.2%)		
Independence(6,7)	2 (4.0%)	35 (70.0%)	3 (14.3%)	17 (81.0%)	2 (9.5%)	11 (52.4%)	13 (9.7%)	126 (94.0%)		
Missing	0 (0.0%)	0 (0.0%)	0 (0.0%)	0 (0.0%)	0 (0.0%)	0 (0.0%)	1 (0.7%)	1 (0.7%)		
**FIM transfer(toilet)**										
complete Dependence(1,2)	0 (0.0%)	0 (0.0%)	0 (0.0%)	0 (0.0%)	0 (0.0%)	0 (0.0%)	0 (0.0%)	0 (0.0%)		0.814
Modified Dependence(3-5)	49 (98.0%)	13 (26.0%)	21 (100.0%)	4 (19.0%)	21 (100.0%)	5 (23.8%)	130 (97.0%)	9 (6.7%)		
Independence(6,7)	1 (2.0%)	37 (74.0%)	0 (0.0%)	17 (81.0%)	0 (0.0%)	16 (76.2%)	3 (2.2%)	124 (92.5%)		
Missing	0 (0.0%)	0 (0.0%)	0 (0.0%)	0 (0.0%)	0 (0.0%)	0 (0.0%)	1 (0.7%)	1 (0.7%)		
**FIM locomotion(walk/wheelchair)**										
complete Dependence(1,2)	10 (20.0%)	4 (8.0%)	4 (19.0%)	0 (0.0%)	11 (52.4%)	4 (19.0%)	56 (41.8%)	13 (9.7%)	*	0.001
Modified Dependence(3-5)	37 (74.0%)	11 (22.0%)	16 (76.2%)	5 (23.8%)	10 (47.6%)	8 (38.1%)	77 (57.5%)	34 (25.4%)		
Independence(6,7)	3 (6.0%)	35 (70.0%)	0 (0.0%)	16 (76.2%)	0 (0.0%)	9 (42.9%)	0 (0.0%)	85 (63.4%)		
Missing	0 (0.0%)	0 (0.0%)	1 (4.8%)	0 (0.0%)	0 (0.0%)	0 (0.0%)	1 (0.7%)	2 (1.5%)		
**FIM comprehension**										
complete Dependence(1,2)	2 (4.0%)	3 (6.0%)	1 (4.8%)	1 (4.8%)	0 (0.0%)	0 (0.0%)	0 (0.0%)	0 (0.0%)		0.093
Modified Dependence(3-5)	31 (62.0%)	20 (40.0%)	12 (57.1%)	6 (28.6%)	18 (85.7%)	14 (66.7%)	97 (72.4%)	41 (30.6%)		
Independence(6,7)	17 (34.0%)	27 (54.0%)	7 (33.3%)	13 (61.9%)	3 (14.3%)	7 (33.3%)	35 (26.1%)	91 (67.9%)		
Missing	0 (0.0%)	0 (0.0%)	1 (4.8%)	1 (4.8%)	0 (0.0%)	0 (0.0%)	2 (1.5%)	2 (1.5%)		
**FIM social interaction**										
complete Dependence(1,2)	1 (2.0%)	1 (2.0%)	0 (0.0%)	0 (0.0%)	1 (4.8%)	0 (0.0%)	3 (2.2%)	2 (1.5%)		0.895
Modified Dependence(3-5)	34 (68.0%)	27 (54.0%)	13 (61.9%)	9 (42.9%)	16 (76.2%)	12 (57.1%)	91 (67.9%)	21 (15.7%)		
Independence(6,7)	15 (30.0%)	22 (44.0%)	7 (33.3%)	11 (52.4%)	4 (19.0%)	9 (42.9%)	38 (28.4%)	109 (81.3%)		
Missing	0 (0.0%)	0 (0.0%)	1 (4.8%)	1 (4.8%)	0 (0.0%)	0 (0.0%)	2 (1.5%)	2 (1.5%)		
**FIM problem solving**										
complete Dependence(1,2)	6 (12.0%)	3 (6.0%)	0 (0.0%)	0 (0.0%)	2 (9.5%)	2 (9.5%)	5 (3.7%)	2 (1.5%)		0.332
Modified Dependence(3-5)	44 (88.0%)	38 (76.0%)	20 (95.2%)	14 (66.7%)	19 (90.5%)	15 (71.4%)	126 (94.0%)	73 (54.5%)		
Independence(6,7)	0 (0.0%)	9 (18.0%)	0 (0.0%)	6 (28.6%)	0 (0.0%)	4 (19.0%)	1 (0.7%)	57 (42.5%)		
Missing	0 (0.0%)	0 (0.0%)	1 (4.8%)	1 (4.8%)	0 (0.0%)	0 (0.0%)	2 (1.5%)	2 (1.5%)		

Abbreviations: FIM; Functional Independence Measure (range, 18–126, a higher score indicated higher independence), OT; Occupational therapist, PT; Physical therapist, ST; Speech Language therapist

Continuous variable, Mean(SD)	Class3 (N=214)									p-value
	Usual care N=49		Self exercise N=8		Hospital-staff training N=39		Both N=118			
**Age**	73.0 (11.6)		73.4 (16.2)		75.4 (10.2)		65.7 (12.8)		*	<0.001
**Length of stay**, days	113.4 (45.7)		122.8 (45.0)		110.9 (41.5)		128.2 (31.2)			0.028
Intervention by PT, OT and ST(units/day)	4.2 (1.6)		6.9 (0.9)		3.6 (1.6)		4.9 (1.7)		*	<0.001
	**admission**	**discharge**	**admission**	**discharge**	**admission**	**discharge**	**admission**	**discharge**		
**Motor-FIM**(admission)	24.6 (7.7)	51.6 (22.6)	29.4 (6.5)	63.0 (13.3)	23.5 (7.9)	48.8 (19.0)	28.7 (9.4)	63.0 (15.4)	*	0.003
**Cognitive-FIM**(admission)	19.1 (4.7)	22.8 (7.2)	19.8 (4.1)	23.6 (5.4)	18.4 (4.6)	21.1 (5.2)	20.6 (4.9)	27.5 (5.0)		0.071
**FIM(total)**(admission)	43.4 (9.0)	73.4 (27.6)	46.5 (9.9)	85.1 (18.6)	42.1 (10.3)	70.1 (22.0)	49.0 (11.3)	89.7 (18.9)	*	0.001
**Category variable, N(%)**										
**Sex** male	24 (49.0%)		2 (25.0%)		13 (33.3%)		53 (44.9%)			0.329
**Age-group**										
≧54	3 (6.1%)		1 (12.5%)		3 (7.7%)		19 (16.1%)		*	0.001
55-64	4 (8.2%)		1 (12.5%)		3 (7.7%)		30 (25.4%)			
65-74	17 (34.7%)		0 (0.0%)		9 (23.1%)		35 (29.7%)			
75-84	17 (34.7%)		5 (62.5%)		16 (41.0%)		31 (26.3%)			
≧85	8 (16.3%)		1 (12.5%)		8 (20.5%)		3 (2.5%)			
Missing	0 (0.0%)		0 (0.0%)		0 (0.0%)		0 (0.0%)			
**Major Stroke Types**										
cerebral infarction	32 (65.3%)		6 (75.0%)		24 (61.5%)		62 (52.5%)			0.562
cerebral hemorrhage	15 (30.6%)		2 (25.0%)		13 (33.3%)		50 (42.4%)			
subarachnoid hemorrhage	1 (2.0%)		0 (0.0%)		2 (5.1%)		5 (4.2%)			
Missing	1 (2.0%)		0 (0.0%)		0 (0.0%)		1 (0.8%)			
**Body side with post-stroke paralysis**										
right	19 (38.8%)		2 (25.0%)		12 (30.8%)		37 (31.4%)			0.215
left	28 (57.1%)		4 (50.0%)		19 (48.7%)		53 (44.9%)			
both	2 (4.1%)		0 (0.0%)		5 (12.8%)		8 (6.8%)			
nothing	0 (0.0%)		1 (12.5%)		2 (5.1%)		1 (0.8%)			
Missing	0 (0.0%)		1 (12.5%)		1 (2.6%)		19 (16.1%)			
**Conducting conferences**										
peliodic	37 (75.5%)		5 (62.5%)		9 (23.1%)		68 (57.6%)		*	<0.001
peliodic and as needed	11 (22.4%)		3 (37.5%)		30 (76.9%)		50 (42.4%)			
Missing	1 (2.0%)		0 (0.0%)		0 (0.0%)		0 (0.0%)			
**Orthotic prescriptions**										
Yes	14 (28.6%)		4 (50.0%)		6 (15.4%)		49 (41.5%)		*	0.001
No	35 (71.4%)		4 (50.0%)		10 (25.6%)		28 (23.7%)			
Missing	0 (0.0%)		0 (0.0%)		23 (59.0%)		41 (34.7%)			
	**admission**	**discharge**	**admission**	**discharge**	**admission**	**discharge**	**admission**	**discharge**		
**FIM eating**										
complete Dependence(1,2)	18 (36.7%)	2 (4.1%)	1 (12.5%)	0 (0.0%)	6 (15.4%)	0 (0.0%)	13 (11.0%)	3 (2.5%)	*	<0.001
Modified Dependence(3-5)	19 (38.8%)	12 (24.5%)	3 (37.5%)	1 (12.5%)	23 (59.0%)	19 (48.7%)	84 (71.2%)	35 (29.7%)		
Independence(6,7)	12 (24.5%)	35 (71.4%)	4 (50.0%)	7 (87.5%)	10 (25.6%)	20 (51.3%)	21 (17.8%)	79 (66.9%)		
Missing	0 (0.0%)	0 (0.0%)	0 (0.0%)	0 (0.0%)	0 (0.0%)	0 (0.0%)	0 (0.0%)	1 (0.8%)		
**FIM toileting**										
complete Dependence(1,2)	45 (91.8%)	20 (40.8%)	6 (75.0%)	1 (12.5%)	35 (89.7%)	15 (38.5%)	112 (94.9%)	12 (10.2%)		0.057
Modified Dependence(3-5)	3 (6.1%)	14 (28.6%)	2 (25.0%)	4 (50.0%)	4 (10.3%)	13 (33.3%)	4 (3.4%)	34 (28.8%)		
Independence(6,7)	0(0%)	15 (30.6%)	0 (0.0%)	3 (37.5%)	0 (0.0%)	11 (28.2%)	0 (0.0%)	71 (60.2%)		
Missing	1 (2.0%)	0 (0.0%)	0 (0.0%)	0 (0.0%)	0 (0.0%)	0 (0.0%)	2 (1.7%)	1 (0.8%)		
**FIM transfer(toilet)**										
complete Dependence(1,2)	38 (77.6%)	10 (20.4%)	6 (75.0%)	0 (0.0%)	34 (87.2%)	9 (23.1%)	75 (63.6%)	3 (2.5%)	*	0.019
Modified Dependence(3-5)	11 (22.4%)	22 (44.9%)	1 (12.5%)	5 (62.5%)	5 (12.8%)	16 (41.0%)	43 (36.4%)	48 (40.7%)		
Independence(6,7)	0 (0.0%)	16 (32.7%)	0 (0.0%)	2 (25.0%)	0 (0.0%)	14 (35.9%)	0 (0.0%)	67 (56.8%)		
Missing	0 (0.0%)	1 (2.0%)	1 (12.5%)	1 (12.5%)	0 (0.0%)	0 (0.0%)	0 (0.0%)	0 (0.0%)		
**FIM locomotion(walk/wheelchair)**										
complete Dependence(1,2)	41 (83.7%)	16 (32.7%)	6 (75.0%)	0 (0.0%)	36 (92.3%)	17 (43.6%)	116 (98.3%)	30 (25.4%)	*	0.025
Modified Dependence(3-5)	7 (14.3%)	17 (34.7%)	1 (12.5%)	3 (37.5%)	3 (7.7%)	16 (41.0%)	1 (0.8%)	49 (41.5%)		
Independence(6,7)	1 (2.0%)	15 (30.6%)	0 (0.0%)	4 (50.0%)	0 (0.0%)	6 (15.4%)	1 (0.8%)	39 (33.1%)		
Missing	0 (0.0%)	1 (2.0%)	1 (12.5%)	1 (12.5%)	0 (0.0%)	0 (0.0%)	0 (0.0%)	0 (0.0%)		
**FIM comprehension**										
complete Dependence(1,2)	7 (14.3%)	2 (4.1%)	0 (0.0%)	0 (0.0%)	3 (7.7%)	2 (5.1%)	8 (6.8%)	0 (0.0%)		0.613
Modified Dependence(3-5)	37 (75.5%)	26 (53.1%)	7 (87.5%)	6 (75.0%)	31 (79.5%)	26 (66.7%)	89 (75.4%)	42 (35.6%)		
Independence(6,7)	5 (10.2%)	20 (40.8%)	1 (12.5%)	2 (25.0%)	5 (12.8%)	11 (28.2%)	20 (16.9%)	73 (61.9%)		
Missing	0 (0.0%)	1 (2.0%)	0 (0.0%)	0 (0.0%)	0 (0.0%)	0 (0.0%)	1 (0.8%)	3 (2.5%)		
**FIM social interaction**										
complete Dependence(1,2)	4 (8.2%)	4 (8.2%)	0 (0.0%)	0 (0.0%)	7 (17.9%)	3 (7.7%)	8 (6.8%)	0 (0.0%)	*	0.022
Modified Dependence(3-5)	28 (57.1%)	23 (46.9%)	7 (87.5%)	4 (50.0%)	29 (74.4%)	27 (69.2%)	86 (72.9%)	36 (30.5%)		
Independence(6,7)	17 (34.7%)	22 (44.9%)	1 (12.5%)	4 (50.0%)	3 (7.7%)	9 (23.1%)	23 (19.5%)	79 (66.9%)		
Missing	0 (0.0%)	0 (0.0%)	0 (0.0%)	0 (0.0%)	0 (0.0%)	0 (0.0%)	1 (0.8%)	3 (2.5%)		
**FIM problem solving**										
complete Dependence(1,2)	21 (42.9%)	19 (38.8%)	3 (37.5%)	1 (12.5%)	11 (28.2%)	10 (25.6%)	29 (24.6%)	11 (9.3%)		0.356
Modified Dependence(3-5)	27 (55.1%)	19 (38.8%)	5 (62.5%)	7 (87.5%)	28 (71.8%)	27 (69.2%)	86 (72.9%)	68 (57.6%)		
Independence(6,7)	1 (2.0%)	11 (22.4%)	0 (0.0%)	0 (0.0%)	0 (0.0%)	2 (5.1%)	2 (1.7%)	36 (30.5%)		
Missing	0 (0.0%)	0 (0.0%)	0 (0.0%)	0 (0.0%)	0 (0.0%)	0 (0.0%)	1 (0.8%)	3 (2.5%)		

Abbreviations: FIM; Functional Independence Measure (range, 18–126, a higher score indicated higher independence), OT; Occupational therapist, PT; Physical therapist, ST; Speech Language therapist

Continuous variable, Mean(SD)	Class4 (N=222) Usual care N=43		Self exercise N=19		Hospital-staff training N=6		Both N=154			p-value
**Age**	68.3 (12.2)		67.0 (10.2)		70.8 (11.1)		63.7 (11.8)			0.069
**Length of stay**, days	110.3 (46.3)		102.4 (37.9)		91.8 (33.0)		101.0 (40.4)			0.545
Intervention by PT, OT and ST(units/day)	5.0 (1.6)		5.6 (1.8)		5.0 (2.4)		4.4 (2.0)			0.024
	**admission**	**discharge**	**admission**	**discharge**	**admission**	**discharge**	**admission**	**discharge**		
**Motor-FIM**(admission)	49.3 (14.1)	74.5 (14.1)	61.3 (10.3)	79.8 (7.5)	38.3 (12.0)	59.5 (23.8)	48.8 (15.4)	76.7 (10.9)	*	0.002
**Cognitive-FIM**(admission)	31.7 (3.3)	32.7 (2.2)	31.3 (2.8)	32.4 (2.9)	29.7 (2.9)	29.3 (4.6)	31.7 (2.6)	33.3 (2.3)		0.333
**FIM(total)**(admission)	80.5 (15.2)	107.2 (15.1)	93.3 (11.5)	112.4 (9.5)	68.0 (12.3)	88.8 (27.9)	80.1 (16.1)	109.8 (12.1)	*	0.001
**Category variable, N(%)**										
**Sex** male	13 (30.2%)		6 (31.6%)		3 (50.0%)		67 (43.5%)			0.347
**Age-group**										
≧54	4 (9.3%)		1 (5.3%)		1 (16.7%)		29 (18.8%)			0.066
55-64	10 (23.3%)		7 (36.8%)		1 (16.7%)		59 (38.3%)			
65-74	12 (27.9%)		7 (36.8%)		1 (16.7%)		30 (19.5%)			
75-84	14 (32.6%)		4 (21.1%)		3 (50.0%)		35 (22.7%)			
≧85	3 (7.0%)		0 (0.0%)		0 (0.0%)		1 (0.6%)			
Missing	0 (0.0%)		0 (0.0%)		0 (0.0%)		0 (0.0%)			
**Major Stroke Types**										
cerebral infarction	26 (60.5%)		11 (57.9%)		4 (66.7%)		89 (57.8%)			0.791
cerebral hemorrhage	16 (37.2%)		6 (31.6%)		2 (33.3%)		58 (37.7%)			
subarachnoid hemorrhage	1 (2.3%)		2 (10.5%)		0 (0.0%)		3 (1.9%)			
Missing	0 (0.0%)		0 (0.0%)		0 (0.0%)		4 (2.5%)			
**Body side with post-stroke paralysis**										
right	9 (20.9%)		5 (26.3%)		1 (16.7%)		59 (38.3%)			0.165
left	28 (65.1%)		10 (52.6%)		5 (83.3%)		67 (43.5%)			
both	1 (2.3%)		1 (5.3%)		0 (0.0%)		10 (6.5%)			
nothing	5 (11.6%)		2 (10.5%)		0 (0.0%)		7 (4.5%)			
Missing	0 (0.0%)		1 (5.3%)		0 (0.0%)		11 (7.1%)			
**Conducting conferences**										
peliodic	32 (74.4%)		15 (78.9%)		1 (16.7%)		58 (37.7%)		*	<0.001
peliodic and as needed	10 (23.3%)		4 (21.1%)		5 (83.3%)		93 (60.4%)			
Missing	1 (2.3%)		0 (0.0%)		0 (0.0%)		3 (1.9%)			
**Orthotic prescriptions**										
Yes	17 (39.5%)		5 (26.3%)		3 (50.0%)		40 (26.0%)			0.391
No	26 (60.5%)		14 (73.7%)		2 (33.3%)		49 (31.8%)			
Missing	0 (0.0%)		0 (0.0%)		1 (16.7%)		65 (42.2%)			
	**admission**	**discharge**	**admission**	**discharge**	**admission**	**discharge**	**admission**	**discharge**		
**FIM eating**										
complete Dependence(1,2)	5 (11.6%)	2 (4.7%)	0 (0.0%)	0 (0.0%)	0 (0.0%)	0 (0.0%)	9 (5.8%)	0 (0.0%)		0.085
Modified Dependence(3-5)	9 (20.9%)	3 (7.0%)	1 (5.3%)	0 (0.0%)	3 (50.0%)	2 (33.3%)	43 (27.9%)	9 (5.8%)		
Independence(6,7)	29 (67.4%)	38 (88.4%)	18 (94.7%)	19 (100.0%)	3 (50.0%)	4 (66.7%)	102 (66.2%)	145 (94.2%)		
Missing	0 (0.0%)	0 (0.0%)	0 (0.0%)	0 (0.0%)	0 (0.0%)	0 (0.0%)	0 (0.0%)	0 (0.0%)		
**FIM toileting**										
complete Dependence(1,2)	13 (30.2%)	2 (4.7%)	2 (10.5%)	0 (0.0%)	3 (50.0%)	1 (16.7%)	50 (32.5%)	5 (3.2%)		0.428
Modified Dependence(3-5)	27 (62.8%)	6 (14.0%)	14 (73.7%)	2 (10.5%)	3 (50.0%)	3 (50.0%)	89 (57.8%)	9 (5.8%)		
Independence(6,7)	3 (7.0%)	35 (81.4%)	3 (15.8%)	17 (89.5%)	0 (0.0%)	2 (33.3%)	15 (9.7%)	140 (90.9%)		
Missing	0 (0.0%)	0 (0.0%)	0 (0.0%)	0 (0.0%)	0 (0.0%)	0 (0.0%)	0 (0.0%)	0 (0.0%)		
**FIM transfer(toilet)**										
complete Dependence(1,2)	13 (30.2%)	0 (0.0%)	0 (0.0%)	0 (0.0%)	1 (16.7%)	1 (16.7%)	25 (16.2%)	2 (1.3%)		0.065
Modified Dependence(3-5)	28 (65.1%)	8 (18.6%)	19 (100.0%)	2 (10.5%)	5 (83.3%)	3 (50.0%)	117 (76.0%)	11 (7.1%)		
Independence(6,7)	2 (4.7%)	35 (81.4%)	0 (0.0%)	17 (89.5%)	0 (0.0%)	2 (33.3%)	12 (7.8%)	141 (91.6%)		
Missing	0 (0.0%)	0 (0.0%)	0 (0.0%)	0 (0.0%)	0 (0.0%)	0 (0.0%)	0 (0.0%)	0 (0.0%)		
**FIM locomotion(walk/wheelchair)**										
complete Dependence(1,2)	26 (60.5%)	2 (4.7%)	2 (10.5%)	0 (0.0%)	5 (83.3%)	2 (33.3%)	102 (66.2%)	37 (24.0%)	*	<0.001
Modified Dependence(3-5)	15 (34.9%)	10 (23.3%)	14 (73.7%)	5 (26.3%)	1 (16.7%)	3 (50.0%)	51 (33.1%)	31 (20.1%)		
Independence(6,7)	2 (4.7%)	31 (72.1%)	3 (15.8%)	14 (73.7%)	0 (0.0%)	1 (16.7%)	1 (0.6%)	86 (55.8%)		
Missing	0 (0.0%)	0 (0.0%)	0 (0.0%)	0 (0.0%)	0 (0.0%)	0 (0.0%)	0 (0.0%)	0 (0.0%)		
**FIM comprehension**										
complete Dependence(1,2)	0 (0.0%)	0 (0.0%)	0 (0.0%)	0 (0.0%)	0 (0.0%)	0 (0.0%)	0 (0.0%)	0 (0.0%)		0.334
Modified Dependence(3-5)	2 (4.7%)	1 (2.3%)	2 (10.5%)	2 (10.5%)	0 (0.0%)	1 (16.7%)	4 (2.6%)	2 (1.3%)		
Independence(6,7)	41 (95.3%)	42 (97.7%)	17 (89.5%)	17 (89.5%)	6 (100.0%)	5 (83.3%)	149 (96.8%)	151 (98.1%)		
Missing	0 (0.0%)	0 (0.0%)	0 (0.0%)	0 (0.0%)	0 (0.0%)	0 (0.0%)	1 (0.6%)	1 (0.6%)		
**FIM social interaction**										
complete Dependence(1,2)	0 (0.0%)	0 (0.0%)	0 (0.0%)	0 (0.0%)	0 (0.0%)	0 (0.0%)	1 (0.6%)	0 (0.0%)		0.901
Modified Dependence(3-5)	2 (4.7%)	3 (7.0%)	0 (0.0%)	0 (0.0%)	0 (0.0%)	1 (16.7%)	3 (1.9%)	2 (1.3%)		
Independence(6,7)	41 (95.3%)	40 (93.0%)	19 (100.0%)	19 (100.0%)	6 (100.0%)	5 (83.3%)	149 (96.8%)	151 (98.1%)		
Missing	0 (0.0%)	0 (0.0%)	0 (0.0%)	0 (0.0%)	0 (0.0%)	0 (0.0%)	1 (0.6%)	1 (0.6%)		
**FIM problem solving**										
complete Dependence(1,2)	0 (0.0%)	0 (0.0%)	1 (5.3%)	0 (0.0%)	0 (0.0%)	0 (0.0%)	4 (2.6%)	0 (0.0%)		0.070
Modified Dependence(3-5)	7 (16.3%)	8 (18.6%)	6 (31.6%)	6 (31.6%)	4 (66.7%)	5 (83.3%)	30 (19.5%)	21 (13.6%)		
Independence(6,7)	36 (83.7%)	35 (81.4%)	12 (63.2%)	13 (68.4%)	2 (33.3%)	1 (16.7%)	119 (77.3%)	132 (85.7%)		
Missing	0 (0.0%)	0 (0.0%)	0 (0.0%)	0 (0.0%)	0 (0.0%)	0 (0.0%)	1 (0.6%)	1 (0.6%)		

Abbreviations: FIM; Functional Independence Measure (range, 18–126, a higher score indicated higher independence), OT; Occupational therapist, PT; Physical therapist, ST; Speech Language therapist

Continuous variable, Mean(SD)	Class5 (N=117)									
	Usual care N=43		Self exercise N=2		Hospital-staff training N=33		Both N=39			p-value
**Age**	73.9 (11.2)		72.5 (10.6)		69.5 (7.4)		67.6 (13.0)			0.073
**Length of stay**, days	117.4 (44.2)		128.0 (31.1)		120.6 (40.7)		130.8 (32.3)			0.469
Intervention by PT, OT and ST(units/day)	4.6 (1.8)		3.2 (3.6)		4.0 (1.5)		5.7 (1.7)		*	0.001
	**admission**	**discharge**	**admission**	**discharge**	**admission**	**discharge**	**admission**	**discharge**		
**Motor-FIM**(admission)	15.4 (3.4)	36.0 (23.1)	18.5 (2.1)	55.0 (43.8)	15.9 (4.9)	35.8 (18.6)	17.7 (5.3)	41.4 (22.1)		0.116
**Cognitive-FIM**(admission)	9.0 (4.0)	14.6 (8.2)	7.5 (3.5)	23.0 (12.7)	7.6 (3.0)	15.3 (6.6)	9.0 (2.6)	16.9 (8.0)		0.227
**FIM(total)**(admission)	24.4 (5.9)	50.4 (29.1)	26.0 (5.7)	78.0 (56.6)	23.5 (7.4)	51.1 (24.1)	26.6 (6.4)	57.7 (27.7)		0.231
**Category variable, N(%)**										
**Sex** male	20 (46.5%)		2 (100.0%)		9 (27.3%)		21 (53.8%)		*	0.048
**Age-group**										
≧54	3 (7.0%)		0 (0.0%)		0 (0.0%)		5 (12.8%)			0.116
55-64	4 (9.3%)		0 (0.0%)		10 (30.3%)		7 (17.9%)			
65-74	12 (27.9%)		1 (50.0%)		11 (33.3%)		13 (33.3%)			
75-84	18 (41.9%)		1 (50.0%)		12 (36.4%)		12 (30.8%)			
≧85	6 (14.0%)		0 (0.0%)		0 (0.0%)		1 (2.6%)			
Missing	0 (0.0%)		0 (0.0%)		0 (0.0%)		1 (2.6%)			
**Major Stroke Types**										
cerebral infarction	29 (67.4%)		0 (0.0%)		17 (51.5%)		21 (53.8%)			0.175
cerebral hemorrhage	10 (23.3%)		1 (50.0%)		12 (36.4%)		16 (41.0%)			
subarachnoid hemorrhage	4 (9.3%)		1 (50.0%)		4 (12.1%)		2 (5.1%)			
Missing	0 (0.0%)		0 (0.0%)		0 (0.0%)		0 (0.0%)			
**Body side with post-stroke paralysis**										
right	24 (55.8%)		2 (100.0%)		13 (39.4%)		26 (66.7%)			0.174
left	17 (39.5%)		0 (0.0%)		15 (45.5%)		11 (28.2%)			
both	2 (4.7%)		0 (0.0%)		3 (9.1%)		0 (0.0%)			
nothing	0 (0.0%)		0 (0.0%)		0 (0.0%)		0 (0.0%)			
Missing	0 (0.0%)		0 (0.0%)		2 (6.1%)		2 (5.1%)			
**Conducting conferences**										
peliodic	31 (72.1%)		1 (50.0%)		8 (24.2%)		25 (64.1%)		*	<0.001
peliodic and as needed	12 (27.9%)		1 (50.0%)		24 (72.7%)		13 (33.3%)			
Missing	0 (0.0%)		0 (0.0%)		1 (3.0%)		1 (2.6%)			
**Orthotic prescriptions**										
Yes	10 (23.3%)		0 (0.0%)		6 (18.2%)		12 (30.8%)			0.299
No	33 (76.7%)		2 (100.0%)		8 (24.2%)		20 (51.3%)			
Missing	0 (0.0%)		0 (0.0%)		19 (57.6%)		7 (17.9%)			
	**admission**	**discharge**	**admission**	**discharge**	**admission**	**discharge**	**admission**	**discharge**		
**FIM eating**										
complete Dependence(1,2)	34 (79.1%)	10 (23.3%)	1 (50.0%)	0 (0.0%)	23 (69.7%)	9 (27.3%)	24 (61.5%)	10 (25.6%)		0.334
Modified Dependence(3-5)	9 (20.9%)	17 (39.5%)	1 (50.0%)	1 (50.0%)	10 (30.3%)	16 (48.5%)	15 (38.5%)	21 (53.8%)		
Independence(6,7)	0 (0.0%)	16 (37.2%)	0 (0.0%)	1 (50.0%)	0 (0.0%)	8 (24.2%)	0 (0.0%)	8 (20.5%)		
Missing	0 (0.0%)	0 (0.0%)	0 (0.0%)	0 (0.0%)	0 (0.0%)	0 (0.0%)	0 (0.0%)	0 (0.0%)		
**FIM toileting**										
complete Dependence(1,2)	43 (100.0%)	27 (62.8%)	2 (100.0%)	1 (50.0%)	33 (100.0%)	18 (54.5%)	39 (100.0%)	21 (53.8%)		-
Modified Dependence(3-5)	0 (0.0%)	10 (23.3%)	0 (0.0%)	0 (0.0%)	0 (0.0%)	11 (33.3%)	0 (0.0%)	11 (28.2%)		
Independence(6,7)	0 (0.0%)	6 (14.0%)	0 (0.0%)	1 (50.0%)	0 (0.0%)	4 (12.1%)	0 (0.0%)	7 (17.9%)		
Missing	0 (0.0%)	0 (0.0%)	0 (0.0%)	0 (0.0%)	0 (0.0%)	0 (0.0%)	0 (0.0%)	0 (0.0%)		
**FIM transfer(toilet)**										
complete Dependence(1,2)	43 (100.0%)	19 (44.2%)	2 (100.0%)	1 (50.0%)	33 (100.0%)	13 (39.4%)	38 (97.4%)	14 (35.9%)		-
Modified Dependence(3-5)	0 (0.0%)	17 (39.5%)	0 (0.0%)	0 (0.0%)	0 (0.0%)	16 (48.5%)	0 (0.0%)	16 (41.0%)		
Independence(6,7)	0 (0.0%)	7 (16.3%)	0 (0.0%)	1 (50.0%)	0 (0.0%)	4 (12.1%)	0 (0.0%)	9 (23.1%)		
Missing	0 (0.0%)	0 (0.0%)	0 (0.0%)	0 (0.0%)	0 (0.0%)	0 (0.0%)	1 (2.6%)	0 (0.0%)		
**FIM locomotion(walk/wheelchair)**										
complete Dependence(1,2)	43 (100.0%)	23 (53.5%)	2 (100.0%)	1 (50.0%)	33 (100.0%)	13 (39.4%)	36 (92.3%)	20 (51.3%)		0.547
Modified Dependence(3-5)	0 (0.0%)	11 (25.6%)	0 (0.0%)	0 (0.0%)	0 (0.0%)	16 (48.5%)	1 (2.6%)	11 (28.2%)		
Independence(6,7)	0 (0.0%)	9 (20.9%)	0 (0.0%)	1 (50.0%)	0 (0.0%)	4 (12.1%)	0 (0.0%)	7 (17.9%)		
Missing	0 (0.0%)	0 (0.0%)	0 (0.0%)	0 (0.0%)	0 (0.0%)	0 (0.0%)	2 (5.1%)	1 (2.6%)		
**FIM comprehension**										
complete Dependence(1,2)	35 (81.4%)	21 (48.8%)	2 (100.0%)	1 (50.0%)	25 (75.8%)	8 (24.2%)	29 (74.4%)	8 (20.5%)		0.796
Modified Dependence(3-5)	7 (16.3%)	17 (39.5%)	0 (0.0%)	0 (0.0%)	8 (24.2%)	22 (66.7%)	9 (23.1%)	20 (51.3%)		
Independence(6,7)	1 (2.3%)	5 (11.6%)	0 (0.0%)	1 (50.0%)	0 (0.0%)	3 (9.1%)	0 (0.0%)	10 (25.6%)		
Missing	0 (0.0%)	0 (0.0%)	0 (0.0%)	0 (0.0%)	0 (0.0%)	0 (0.0%)	1 (2.6%)	1 (2.6%)		
**FIM social interaction**										
complete Dependence(1,2)	38 (88.4%)	20 (46.5%)	1 (50.0%)	0 (0.0%)	30 (90.9%)	13 (39.4%)	33 (84.6%)	15 (38.5%)		0.306
Modified Dependence(3-5)	2 (4.7%)	13 (30.2%)	1 (50.0%)	1 (50.0%)	2 (6.1%)	17 (51.5%)	4 (10.3%)	15 (38.5%)		
Independence(6,7)	3 (7.0%)	10 (23.3%)	0 (0.0%)	1 (50.0%)	1 (3.0%)	3 (9.1%)	1 (2.6%)	8 (20.5%)		
Missing	0 (0.0%)	0 (0.0%)	0 (0.0%)	0 (0.0%)	0 (0.0%)	0 (0.0%)	1 (2.6%)	1 (2.6%)		
**FIM problem solving**										
complete Dependence(1,2)	43 (100.0%)	31 (72.1%)	2 (100.0%)	1 (50.0%)	33 (100.0%)	23 (69.7%)	38 (97.4%)	22 (56.4%)		-
Modified Dependence(3-5)	0 (0.0%)	9 (20.9%)	0 (0.0%)	0 (0.0%)	0 (0.0%)	9 (27.3%)	0 (0.0%)	12 (30.8%)		
Independence(6,7)	0 (0.0%)	3 (7.0%)	0 (0.0%)	1 (50.0%)	0 (0.0%)	1 (3.0%)	0 (0.0%)	4 (10.3%)		
Missing	0 (0.0%)	0 (0.0%)	0 (0.0%)	0 (0.0%)	0 (0.0%)	0 (0.0%)	1 (2.6%)	1 (2.6%)		

Abbreviations: FIM; Functional Independence Measure (range, 18–126, a higher score indicated higher independence), OT; Occupational therapist, PT; Physical therapist, ST; Speech Language therapist

Continuous variable, Mean(SD)	Class6 (N=98) Usual care N=13		Self exercise N=12		Hospital-staff training N=10		Both N=63		p-value
**Age**	66.3 (12.1)		56.5 (14.4)		63.8 (22.4)		63.8 (13.6)		0.358
**Length of stay**, days	85.2 (36.5)		82.3 (41.0)		84.5 (37.1)		81.3 (36.9)		0.985
Intervention by PT, OT and ST(units/day)	5.2 (1.3)		5.9 (1.4)		3.4 (1.6)		4.9 (1.8)		* 0.007
	**admission**	**discharge**	**admission**	**discharge**	**admission**	**discharge**	**admission**	**discharge**	
**Motor-FIM**(admission)	77.5 (11.2)	84.5 (5.6)	78.2 (9.4)	86.7 (5.3)	74.3 (7.8)	84.3 (3.7)	72.4 (9.7)	82.5 (6.7)	0.133
**Cognitive-FIM**(admission)	20.7 (4.3)	22.8 (4.2)	22.0 (5.1)	27.4 (4.6)	20.2 (6.1)	25.2 (5.5)	22.2 (5.3)	27.5 (5.1)	0.588
**FIM(total)**(admission)	98.2 (10.1)	107.2 (7.4)	100.2 (10.5)	114.1 (7.4)	94.5 (11.2)	109.5 (7.8)	94.5 (11.0)	109.9 (9.8)	0.296
**Category variable, N(%)**									
**Sex** male	5 (38%)		3 (25%)		4 (40%)		22 (35%)		0.872
**Age-group**									
≧54	1 (8%)		4 (33%)		2 (20%)		13 (21%)		0.466
55-64	4 (31%)		4 (33%)		2 (20%)		18 (29%)		
65-74	4 (31%)		4 (33%)		1 (10%)		14 (22%)		
75-84	4 (31%)		0 (0%)		4 (40%)		17 (27%)		
≧85	0 (0.0%)		0 (0.0%)		1 (10%)		1 (2%)		
Missing	0 (0.0%)		0 (0.0%)		0 (0.0%)		0 (0.0%)		
**Major Stroke Types**									
cerebral infarction	7 (54%)		2 (17%)		5 (50%)		37 (59%)		0.066
cerebral hemorrhage	2 (15%)		6 (50%)		3 (30%)		19 (30%)		
subarachnoid hemorrhage	3 (23%)		4 (33%)		2 (20%)		7 (11%)		
Missing	1 (8%)		0 (0.0%)		0 (0.0%)		0 (0.0%)		
**Body side with post-stroke paralysis**									
right	6 (46%)		7 (58%)		4 (40%)		38 (60%)		0.389
left	3 (23%)		3 (25%)		1 (10%)		8 (13%)		
both	1 (8%)		0 (0.0%)		0 (0.0%)		1 (2%)		
nothing	2 (15%)		2 (17%)		5 (50%)		11 (17%)		
Missing	1 (8%)		0 (0.0%)		0 (0.0%)		5 (8%)		
**Conducting conferences**									
peliodic	10 (77%)		5 (42%)		5 (50%)		41 (65%)		0.448
peliodic and as needed	3 (23%)		5 (42%)		5 (50%)		22 (35%)		
Missing	0 (0.0%)		2 (17%)		0 (0.0%)		0 (0.0%)		
**Orthotic prescriptions**									
Yes	0 (0%)		1 (8%)		0 (0.0%)		4 (6%)		0.613
No	13 (100%)		11 (92%)		7 (70%)		43 (68%)		
Missing	0 (0.0%)		0 (0.0%)		3 (30%)		16 (25%)		
	**admission**	**discharge**	**admission**	**discharge**	**admission**	**discharge**	**admission**	**discharge**	
**FIM eating**									
complete Dependence(1,2)	1 (8%)	0 (0.0%)	0 (0.0%)	0 (0.0%)	0 (0.0%)	0 (0.0%)	1 (2%)	0 (0.0%)	0.336
Modified Dependence(3-5)	0 (0.0%)	0 (0%)	1 (8%)	0 (0%)	0 (0.0%)	0 (0%)	10 (16%)	1 (2%)	
Independence(6,7)	12 (92%)	13 (100%)	11 (92%)	12 (100%)	10 (100%)	10 (100%)	52 (83%)	62 (98%)	
Missing	0 (0.0%)	0 (0.0%)	0 (0.0%)	0 (0.0%)	0 (0.0%)	0 (0.0%)	0 (0.0%)	0 (0.0%)	
**FIM toileting**									
complete Dependence(1,2)	0 (0.0%)	0 (0.0%)	0 (0.0%)	0 (0.0%)	0 (0.0%)	0 (0.0%)	0 (0.0%)	0 (0.0%)	0.354
Modified Dependence(3-5)	2 (15%)	0 (0%)	1 (8%)	0 (0%)	1 (10%)	0 (0%)	2 (3%)	1 (2%)	
Independence(6,7)	11 (85%)	13 (100%)	11 (92%)	12 (100%)	9 (90%)	10 (100%)	61 (97%)	62 (98%)	
Missing	0 (0.0%)	0 (0.0%)	0 (0.0%)	0 (0.0%)	0 (0.0%)	0 (0.0%)	0 (0.0%)	0 (0.0%)	
**FIM transfer(toilet)**									
complete Dependence(1,2)	0 (0.0%)	0 (0.0%)	0 (0.0%)	0 (0.0%)	0 (0.0%)	0 (0.0%)	0 (0.0%)	0 (0.0%)	0.065
Modified Dependence(3-5)	0 (0%)	0 (0.0%)	1 (8%)	0 (0.0%)	0 (0.0%)	0 (0.0%)	0 (0.0%)	0 (0.0%)	
Independence(6,7)	13 (100%)	13 (100%)	11 (92%)	12 (100%)	10 (100%)	10 (100%)	63 (100%)	63 (100%)	
Missing	0 (0.0%)	0 (0.0%)	0 (0.0%)	0 (0.0%)	0 (0.0%)	0 (0.0%)	0 (0.0%)	0 (0.0%)	
**FIM locomotion(walk/wheelchair)**									
complete Dependence(1,2)	0 (0.0%)	0 (0.0%)	1 (8%)	0 (0.0%)	3 (30%)	3 (30%)	16 (25%)	14 (22%)	0.362
Modified Dependence(3-5)	3 (23%)	0 (0.0%)	4 (33%)	1 (8%)	2 (20%)	1 (10%)	13 (21%)	3 (5%)	
Independence(6,7)	10 (77%)	13 (100%)	7 (58%)	11 (92%)	5 (50%)	6 (60%)	34 (54%)	46 (73%)	
Missing	0 (0.0%)	0 (0.0%)	0 (0.0%)	0 (0.0%)	0 (0.0%)	0 (0.0%)	0 (0.0%)	0 (0.0%)	
**FIM comprehension**									
complete Dependence(1,2)	2 (15%)	1 (8%)	3 (25%)	1 (8%)	3 (30%)	1 (10%)	6 (10%)	3 (5%)	0.313
Modified Dependence(3-5)	9 (69%)	7 (54%)	6 (50%)	5 (42%)	7 (70%)	6 (60%)	41 (65%)	18 (29%)	
Independence(6,7)	2 (15%)	5 (38%)	3 (25%)	6 (50%)	0 (0.0%)	3 (30%)	16 (25%)	42 (67%)	
Missing	0 (0.0%)	0 (0.0%)	0 (0.0%)	0 (0.0%)	0 (0.0%)	0 (0.0%)	0 (0.0%)	0 (0.0%)	
**FIM social interaction**									
complete Dependence(1,2)	1 (8%)	0 (0.0%)	0 (0.0%)	0 (0.0%)	2 (20%)	1 (10%)	5 (8%)	0 (0.0%)	0.699
Modified Dependence(3-5)	9 (69%)	8 (62%)	10 (83%)	2 (17%)	6 (60%)	3 (30%)	49 (78%)	27 (43%)	
Independence(6,7)	3 (23%)	5 (38%)	2 (17%)	10 (83%)	2 (20%)	6 (60%)	9 (14%)	36 (57%)	
Missing	0 (0.0%)	0 (0.0%)	0 (0.0%)	0 (0.0%)	0 (0.0%)	0 (0.0%)	0 (0.0%)	0 (0.0%)	
**FIM problem solving**									
complete Dependence(1,2)	2 (15%)	1 (8%)	0 (0.0%)	0 (0.0%)	3 (30%)	1 (10%)	10 (16%)	3 (5%)	0.278
Modified Dependence(3-5)	11 (85%)	12 (92%)	12 (100%)	6 (50%)	7 (70%)	5 (50%)	53 (84%)	41 (65%)	
Independence(6,7)	0 (0.0%)	0 (0.0%)	0 (0.0%)	6 (50%)	0 (0.0%)	4 (40%)	0 (0.0%)	19 (30%)	
Missing	0 (0.0%)	0 (0.0%)	0 (0.0%)	0 (0.0%)	0 (0.0%)	0 (0.0%)	0 (0.0%)	0 (0.0%)	

Abbreviations: FIM; Functional Independence Measure (range, 18–126, a higher score indicated higher independence), OT; Occupational therapist, PT; Physical therapist, ST; Speech Language therapist

Continuous variable, Mean(SD)	Class7 (N=82)									p-value
	Usual care N=28		Self exercise N=4		Hospital-staff training N=15		Both N=35			
**Age**	71.7 (12.2)		73.0 (7.0)		71.7 (11.5)		70.9 (10.5)			0.981
**Length of stay**, days	113.1 (45.3)		109.0 (29.3)		97.4 (28.2)		101.4 (39.9)			0.565
Intervention by PT, OT and ST(units/day)	5.2 (2.0)		5.8 (1.8)		4.0 (1.5)		4.9 (2.0)			0.165
	**admission**	**discharge**	**admission**	**discharge**	**admission**	**discharge**	**admission**	**discharge**		
**Motor-FIM**(admission)	44.6 (12.5)	64.3 (18.7)	49.2 (18.1)	73.8 (5.1)	39.3 (16.6)	63.5 (16.8)	40.2 (11.3)	65.8 (17.2)		0.314
**Cognitive-FIM**(admission)	12.2 (3.5)	17.8 (7.0)	11.5 (2.5)	18.0 (9.4)	12.0 (3.8)	19.0 (4.4)	13.0 (4.0)	20.4 (8.0)		0.740
**FIM(total)**(admission)	56.9 (13.9)	82.1 (23.3)	60.8 (16.1)	91.8 (6.2)	51.3 (19.5)	82.5 (18.9)	52.6 (13.3)	85.2 (23.6)		0.455
**Category variable, N(%)**										
**Sex** male	10 (36%)		0 (0.0%)		3 (20%)		11 (31%)			0.404
**Age-group**										
≧54	3 (11%)		0 (0.0%)		2 (13%)		1 (3%)			0.891
55-64	2 (7%)		0 (0.0%)		1 (7%)		4 (11%)			
65-74	9 (32%)		2 (50%)		4 (27%)		17 (49%)			
75-84	13 (46%)		2 (50%)		7 (47%)		12 (34%)			
≧85	1 (4%)		0 (0%)		1 (7%)		1 (3%)			
Missing	0 (0.0%)		0 (0.0%)		0 (0.0%)		0 (0.0%)			
**Major Stroke Types**										
cerebral infarction	16 (57%)		4 (100%)		10 (67%)		21 (60%)			0.471
cerebral hemorrhage	11 (39%)		0 (0.0%)		3 (20%)		10 (29%)			
subarachnoid hemorrhage	1 (4%)		0 (0.0%)		2 (13%)		4 (11%)			
Missing	0 (0.0%)		0 (0.0%)		0 (0.0%)		0 (0.0%)			
**Body side with post-stroke paralysis**										
right	22 (79%)		3 (75%)		8 (53%)		21 (60%)			0.340
left	4 (14%)		1 (25%)		3 (20%)		5 (14%)			
both	0 (0.0%)		0 (0.0%)		4 (27%)		4 (11%)			
nothing	2 (7%)		0 (0.0%)		0 (0.0%)		2 (6%)			
Missing	0 (0.0%)		0 (0.0%)		0 (0.0%)		3 (9%)			
**Conducting conferences**										
peliodic	19 (68%)		4 (100%)		3 (20%)		22 (63%)		*	0.004
peliodic and as needed	9 (32%)		0 (0.0%)		12 (80%)		13 (37%)			
Missing	0 (0.0%)		0 (0.0%)		0 (0.0%)		0 (0.0%)			
**Orthotic prescriptions**										
Yes	9 (32%)		1 (25%)		2 (13%)		8 (23%)			0.990
No	19 (68%)		3 (75%)		5 (33%)		19 (54%)			
Missing	0 (0.0%)		0 (0.0%)		8 (53%)		8 (23%)			
	**admission**	**discharge**	**admission**	**discharge**	**admission**	**discharge**	**admission**	**discharge**		
**FIM eating**										
complete Dependence(1,2)	7 (25%)	0 (0%)	0 (0.0%)	0 (0.0%)	1 (7%)	1 (7%)	5 (14%)	2 (6%)		0.463
Modified Dependence(3-5)	14 (50%)	7 (25%)	2 (50%)	0 (0.0%)	10 (67%)	6 (40%)	23 (66%)	12 (34%)		
Independence(6,7)	7 (25%)	21 (75%)	2 (50%)	4 (100%)	4 (27%)	8 (53%)	6 (17%)	20 (57%)		
Missing	0 (0.0%)	0 (0.0%)	0 (0.0%)	0 (0.0%)	0 (0.0%)	0 (0.0%)	1 (3%)	1 (3%)		
**FIM toileting**										
complete Dependence(1,2)	7 (25%)	2 (7%)	0 (0.0%)	0 (0.0%)	7 (47%)	1 (7%)	12 (34%)	2 (6%)		0.110
Modified Dependence(3-5)	20 (71%)	13 (46%)	3 (75%)	1 (25%)	7 (47%)	5 (33%)	22 (63%)	11 (31%)		
Independence(6,7)	1 (4%)	13 (46%)	1 (25%)	3 (75%)	1 (7%)	9 (60%)	0 (0%)	21 (60%)		
Missing	0 (0.0%)	0 (0.0%)	0 (0.0%)	0 (0.0%)	0 (0.0%)	0 (0.0%)	1 (3%)	1 (3%)		
**FIM transfer(toilet)**										
complete Dependence(1,2)	27 (96%)	0 (0.0%)	4 (100%)	0 (0.0%)	15 (100%)	0 (0.0%)	31 (89%)	0 (0.0%)		0.350
Modified Dependence(3-5)	1 (4%)	15 (54%)	0 (0.0%)	0 (0.0%)	0 (0.0%)	6 (40%)	4 (11%)	11 (31%)		
Independence(6,7)	0 (0.0%)	13 (46%)	0 (0.0%)	4 (100%)	0 (0.0%)	9 (60%)	0 (0.0%)	24 (69%)		
Missing	0 (0.0%)	0 (0.0%)	0 (0.0%)	0 (0.0%)	0 (0.0%)	0 (0.0%)	0 (0.0%)	0 (0.0%)		
**FIM locomotion(walk/wheelchair)**										
complete Dependence(1,2)	11 (39%)	4 (14%)	1 (25%)	0 (0.0%)	9 (60%)	5 (33%)	20 (57%)	5 (14%)		0.551
Modified Dependence(3-5)	15 (54%)	12 (43%)	3 (75%)	0 (0.0%)	6 (40%)	6 (40%)	14 (40%)	18 (51%)		
Independence(6,7)	2 (7%)	12 (43%)	0 (0.0%)	4 (100%)	0 (0.0%)	4 (27%)	1 (3%)	12 (34%)		
Missing	0 (0.0%)	0 (0.0%)	0 (0.0%)	0 (0.0%)	0 (0.0%)	0 (0.0%)	0 (0.0%)	0 (0.0%)		
**FIM comprehension**										
complete Dependence(1,2)	19 (68%)	10 (36%)	3 (75%)	1 (25%)	6 (40%)	1 (7%)	9 (26%)	8 (23%)	*	0.035
Modified Dependence(3-5)	9 (32%)	14 (50%)	1 (25%)	2 (50%)	9 (60%)	14 (93%)	25 (71%)	16 (46%)		
Independence(6,7)	0 (0.0%)	4 (14%)	0 (0.0%)	1 (25%)	0 (0.0%)	0 (0.0%)	1 (3%)	11 (31%)		
Missing	0 (0.0%)	0 (0.0%)	0 (0.0%)	0 (0.0%)	0 (0.0%)	0 (0.0%)	0 (0.0%)	0 (0.0%)		
**FIM social interaction**										
complete Dependence(1,2)	19 (68%)	11 (39%)	4 (100%)	2 (50%)	11 (73%)	1 (7%)	23 (66%)	7 (20%)		0.851
Modified Dependence(3-5)	5 (18%)	10 (36%)	0 (0.0%)	1 (25%)	3 (20%)	10 (67%)	8 (23%)	17 (49%)		
Independence(6,7)	4 (14%)	7 (25%)	0 (0.0%)	1 (25%)	1 (7%)	4 (27%)	4 (11%)	11 (31%)		
Missing	0 (0.0%)	0 (0.0%)	0 (0.0%)	0 (0.0%)	0 (0.0%)	0 (0.0%)	0 (0.0%)	0 (0.0%)		
**FIM problem solving**										
complete Dependence(1,2)	26 (93%)	14 (50%)	3 (75%)	1 (25%)	15 (100%)	6 (40%)	33 (94%)	10 (29%)		0.386
Modified Dependence(3-5)	1 (4%)	12 (43%)	1 (25%)	2 (50%)	0 (0.0%)	9 (60%)	2 (6%)	22 (63%)		
Independence(6,7)	1 (4%)	2 (7%)	0 (0.0%)	1 (25%)	0 (0.0%)	0 (0%)	0 (0.0%)	3 (9%)		
Missing	0 (0.0%)	0 (0.0%)	0 (0.0%)	0 (0.0%)	0 (0.0%)	0 (0.0%)	0 (0.0%)	0 (0.0%)		

Abbreviations: FIM; Functional Independence Measure (range, 18–126, a higher score indicated higher independence), OT; Occupational therapist, PT; Physical therapist, ST; Speech Language therapist
